# Autonomous cyber-physical security middleware for IoT: anomaly detection and adaptive response in hybrid environments

**DOI:** 10.3389/frai.2025.1675132

**Published:** 2025-12-17

**Authors:** Iván Ortiz-Garcés, William Villegas-Ch, Sergio Luján-Mora

**Affiliations:** 1 Escuela de Ingeniería en Ciberseguridad, Universidad de Las Américas, Quito, Ecuador; 2 Departamento de Lenguajes y Sistemas Informáticos, Universidad de Alicante, Alicante, Spain

**Keywords:** anomaly detection and response, cyber-physical security, hybrid evaluation framework, IoT middleware architecture, artificial intelligence (AI)

## Abstract

The rapid adoption of Internet of Things (IoT) devices in cyber-physical systems introduces significant security challenges, particularly in distributed and heterogeneous environments where operational resilience and real-time threat response are critical. Previous efforts have explored lightweight encryption and modular authentication. Still, few solutions provide a unified framework that integrates real-time anomaly detection, automated mitigation, and performance evaluation under hybrid experimental conditions. This work presents an autonomous multi-layered security architecture for IoT networks, implemented through microservices-based middleware with native support for detection and adaptive response mechanisms. The architecture integrates lightweight anomaly inference models, based on entropy metrics and anomaly scores, with a rule-based engine that executes dynamic containment actions such as node isolation, channel reconfiguration, and key rotation. The system runs on edge hardware (Raspberry Pi, sensors, actuators) and is validated in a hybrid testbed with NS-3 simulations. Experimental results show an F1-Score of 0.931 in physical deployments and 0.912 in simulated scenarios, with anomaly detection latencies below 130 ms and containment actions triggered within 300 ms. Under high-load conditions, CPU usage remains under 60 % and memory consumption below 300 MB. Compared to representative middleware platforms such as BlendSM-DDM and Claimsware, the proposed system uniquely integrates detection, response, and auditability, achieving high scalability and resilience for IoT deployments in real-world hybrid environments.

## 1 Introduction

Internet of Things (IoT) systems have become a core technological infrastructure in industrial automation, smart cities, autonomous transportation, and distributed energy grids (Gligoric et al., 2024). These systems, composed of interconnected devices that collect, process, and transmit data in real time, rely on cyber-physical architectures that are increasingly exposed to security threats. Vulnerabilities in communication, authentication, data integrity, and control mechanisms can compromise not only system functionality but also the physical safety of critical infrastructures. The risk grows in heterogeneous environments where multiple nodes, protocols, and manufacturers coexist without unified protection, which facilitates attacks such as spoofing, denial-of-service (DoS), and traffic manipulation (Ali et al., 2023).

Although several approaches address IoT security, most target isolated mechanisms, such as lightweight encryption, token-based authentication, or blockchain-based traceability (Mahbub, 2021); however, few provide a functional model that integrates anomaly detection, automated countermeasures, and quantitative validation under hybrid operating conditions. Prior efforts, such as Ahmed et al. (2025) and Asif et al. (2022), have shown that hybrid or scalable evaluation frameworks are feasible by combining physical experimentation with simulated environments. Nevertheless, these works remain limited to detection-centric schemes or large-scale distributed processing models, without incorporating modular middleware architectures and real-time adaptive responses. Other middleware-oriented solutions, such as VIRTUS or Endler et al., succeed in enabling secure interoperability through TLS, SASL, and multiprotocol support (MQTT, CoAP, HTTP) (Paris et al., 2023). Still, they primarily ensure communication security without embedding anomaly inference or adaptive response. Similarly, Plazas Olaya et al. (2024) report high detection accuracy in microservice-based IDS deployments, yet their latencies for DoS and slow-DoS detection (0.82 s and 18.74 s, respectively) remain incompatible with stringent IIoT real-time demands. The OC-ASAE framework by Ayad et al. (2025) achieves near-perfect accuracy on benchmark datasets, but its scope is limited to anomaly detection without modular orchestration or containment mechanisms. This landscape confirms that, despite progress, no existing framework simultaneously integrates hybrid validation, anomaly detection, and automated real-time response in a deployable middleware solution, a gap directly addressed by the architecture proposed in this work.

This study introduces a multi-layered cyber-physical security architecture for IoT, implemented through microservices-based middleware. The architecture separates three functional planes: the physical plane (sensors and actuators), the control plane (event management and communication), and the security plane (anomaly detection and adaptive response). Specialized modules for authentication, encryption, auditing, and anomaly inference–based on entropy metrics and anomaly scoring–support adaptive response policies such as node isolation, channel blocking, and key rotation.

The system leverages asynchronous communication technologies (e.g., MQTT), lightweight frameworks (e.g., Flask), and Docker-based microservices for deployment on both edge nodes and virtualized platforms (Paris et al., 2023). Validation is performed in a hybrid environment combining physical devices (Raspberry Pi, sensors, actuators) with NS-3 simulations that reproduce regular traffic, spoofing, flooding, and DoS attacks.

Preliminary results show that the proposed architecture achieves F1-scores above 0.93 with anomaly detection latencies below 130 ms and containment actions within 300 ms. Compared to existing IoT security frameworks, including the microservices-based IDS proposed by Plazas Olaya et al. (2024), which reports F1-scores above 98 % and detection latencies under 20 s in real microservices deployments, and the fog-empowered anomaly detection framework by Ayad et al. (2025), which achieves near-perfect accuracy and F1 values on BoT-IoT and IoT-23 datasets, our approach uniquely integrates detection, automated response, and auditability into a single middleware platform. Unlike previous models that focus primarily on detection, the proposed architecture extends protection by executing containment actions with latencies below 300 ms, validated in both physical and simulated environments. This integration contributes to the design of resilient cyber-physical architectures for complex IoT environments.

The main contribution of this work resides not in a single isolated component, but in the empirical integration of three elements that, to the best of our knowledge, have not been combined in previous proposals: (i) a multi-layered microservice architecture that provides modularity and scalability; (ii) an anomaly detection engine enhanced with entropy-based inference and rigorously validated with optimized hyperparameters; and (iii) an adaptive response mechanism capable of executing containment actions in less than 300 ms. Unlike existing frameworks that focus solely on detection or limit their validation to simulated conditions, our proposal consolidates detection, inference, and automated response into a single middleware platform validated in both physical and controlled environments. This hybrid evaluation, together with reproducible performance metrics (F1-score, latency, CPU/RAM usage, entropy variability), positions the system as a practical and verifiable framework that bridges the gap between academic prototypes and industrial-grade cyber-physical security solutions for heterogeneous IoT networks.

The paper is structured as follows: Section II reviews related work. Section III details the methodology, including the proposed architecture, middleware modules, and evaluation environment. Section IV presents quantitative results on performance, automated response, load efficiency, and attack analysis. Section V discusses the findings in relation to the state of the art, limitations, and practical implications. Finally, Section VI concludes with the contributions and outlines future directions for scalable and autonomous IoT security systems.

## 2 Literature review

The evolution of IoT has driven rapid growth in connected devices that operate across highly heterogeneous physical and virtual environments. This trend has created a pressing need for platforms that ensure cyber-physical security by protecting digital assets while safeguarding the integrity of the physical domain (Goumopoulos, 2024). Integrated security platforms rely on complex middleware architectures that coordinate multiple defense layers, including authentication, encryption, anomaly detection, and automated response mechanisms.

The literature consistently identifies middleware as a key component linking application layers with physical devices in IoT systems. Middleware manages secure communication, data abstraction, and device heterogeneity. Several studies have proposed architectures based on service-oriented models (SOA) or microservices to achieve these objectives. Farooq et al. (2023), for instance, introduce a fused machine learning approach for intrusion detection that demonstrates how combining multiple learning models improves detection robustness in dynamic IoT environments. Their contribution emphasizes that beyond architectural interoperability, the integration of advanced anomaly detection mechanisms is essential to address evolving cyber threats.

The model proposed by Plazas Olaya et al. (2024) exemplifies a microservices-based approach that integrates anomaly detection tasks directly into the middleware. Its implementation was validated in real microservices deployments handling live IoT traffic, reporting high detection accuracy (above 98 %) and F1-scores close to 99 %. Additionally, it demonstrated resilience against DoS and slow DoS attacks, with detection latencies of 0.82 s and 18.74 s, respectively. These results highlight its adaptability and efficiency in heterogeneous IoT environments.

ContextNet middleware extends this concept by supporting authentication, role-based access control, and distributed context reasoning. Its design integrates semantic context modeling, scalable data distribution, and event-driven rationale, making it suitable for collaborative IoT scenarios at scale (Endler et al., 2023). Similarly, Ali et al. (2024b) propose an ensemble and gossip learning-based framework for intrusion detection in vehicle-to-everything (V2X) communication environments. This approach emphasizes collaborative learning, scalability, and robustness in detecting denial-of-service attacks across both infrastructure-based and infrastructureless vehicular networks.

Security mechanisms in IoT middleware are typically organized in layers. The most common include strong authentication and authorization, encryption in transit, message integrity, and auditing. Nasir et al. (2023) introduce a federated machine learning-based intrusion detection approach that enhances these mechanisms by enabling distributed anomaly detection across heterogeneous IoT nodes without centralizing sensitive data. This design not only preserves privacy but also strengthens scalability and resilience, demonstrating how advanced IDS techniques can be embedded into middleware platforms to address resource-constrained environments.

Recent approaches have also focused on enhancing intrusion detection through explainable artificial intelligence. Ali et al. (2024a) propose a detection framework for DoS attacks in autonomous vehicle systems, integrating explainable AI to provide transparent decision-making in anomaly classification. Their results demonstrate improved detection accuracy and interpretability, highlighting the relevance of combining advanced machine learning models with explainability mechanisms to increase trust and robustness in safety-critical IoT applications. Similarly, Endler et al. (2023) present middleware supporting multiple application-layer protocols such as MQTT, CoAP, and HTTP. Their empirical evaluation demonstrates improvements in interoperability, scalability, and device control, while explicitly addressing security gaps observed in earlier frameworks.

Although middleware such as VIRTUS and the ContextNet framework by Endler et al. succeed in enabling secure interoperability through TLS, SASL, and multiprotocol support (MQTT, CoAP, HTTP), these solutions remain limited to communication security. Despite their valuable contributions, frameworks such as VIRTUS and the middleware proposed by Endler et al. (2023) remain insufficient to address the broader challenges of cyber-physical security in heterogeneous IoT networks. Their architecture primarily ensures secure interoperability through TLS, SASL, and multiprotocol support, focusing on communication confidentiality and integrity. However, they lack integrated anomaly detection modules, do not incorporate adaptive response policies capable of mitigating attacks in real time, and provide limited validation under hybrid conditions that combine physical deployments with simulated environments. These limitations prevent their direct applicability in industrial IoT scenarios where low-latency detection and containment, auditability, and resilience against dynamic threats are required. In contrast, the middleware proposed in this work extends beyond secure communication by embedding detection, inference, and automated response mechanisms directly into the architecture, bridging the gap between secure interoperability and proactive, real-time defense.

More recent studies explore emerging technologies to reinforce cyber-physical security. Qadir and Hashmy (2024) propose a multi-level signature verification system using smart contracts on a permissioned blockchain. This design, implemented with IoT-Solidity and integrated with MetaMask and Ganache, achieves efficient distributed verification while reducing human intervention. El-Hajj et al. (2024) combines an intrusion detection system (IDS) with a digital twin built in Eclipse Ditto, enabling real-time detection of attacks such as Hping3 and NMAP. Results indicate improved visibility of system behavior and anticipation of vulnerabilities. Saheed et al. (2025) introduce an explainable deep neural network model (CPS-IoT-PPDNN) that leverages SHAP for interpretability in anomaly detection. Evaluated on Edge-IIoTset and X-IIoTID, the model achieved accuracy above 99.98 % in multi-category scenarios, reducing false positives and improving system reliability.

Hybrid validation that combines simulated and experimental environments remains essential. The model by Plazas Olaya et al. (2024) exemplifies this approach, as it was evaluated using real microservices deployments with live IoT traffic while also incorporating controlled scenarios of DoS and slow DoS attacks. This setup enabled the reporting of quantitative results such as F1-scores above 98 % and detection latencies ranging from 0.82 s to 18.74 s, providing a robust basis for assessing anomaly detection in heterogeneous IoT networks. At the same time, the El-Hajj framework (El-Hajj et al., 2024) implements real attacks on Raspberry Pi devices to assess system resilience under stress.

Evaluations in this domain typically measure precision, accuracy, recall, F1-score, latency, CPU consumption, and blockchain-specific indicators such as throughput and gas limit (Qadir and Hashmy, 2024). These metrics provide objective criteria for comparing models and guiding deployment in production environments.

Despite these advances, challenges persist. Middleware architectures still lack universal security standards, automated service discovery, and privacy-preserving mechanisms for large-scale distributed scenarios. Champaneria et al. (2023) note that migrating to microservices enhances adaptability but raises new concerns regarding coordination, monitoring, and security. Progress is also required in the integration of secure enclaves (e.g., ARM TrustZone) and software-defined networking (SDN), which could support dynamic and segmented responses to cyber threats (Endler et al., 2023; Alghazzawi et al., 2021).

Table 1 summarizes the main middleware-based architectures for IoT security, the technologies they employ, and the environments and metrics used in their evaluations. This synthesis highlights that most proposals focus on isolated aspects or lack hybrid validation, underscoring the research gap that motivates this study.

**Table 1:** Comparison of cyber-physical security architecture and middleware for IoT.

**Reference**	**Architecture or middleware**	**Security technologies**	**Evaluation/metrics**
Farooq et al. (2023)	Fused ML-based IDS	Ensemble of multiple machine learning models for anomaly detection	Accuracy and robustness evaluation in dynamic IoT environments
Plazas Olaya et al. (2024)	Microservices-based IDS	ML-based anomaly detection	F1 = 98.08 % (DT), F1 = 99.85 % (RF slow DoS), Accuracy = 99.62 %; real microservices deployment with IoT traffic
Endler et al. (2023)	ContextNet (TLS, SASL, multiprotocol MQTT/CoAP/HTTP)	Access Control, Authentication, Interoperability	Interoperability validated; lacks anomaly detection and real-time adaptive response
Nasir et al. (2023)	Federated ML-based IDS middleware	Privacy-preserving anomaly detection, scalability, resilience	Validation on distributed IoT datasets with federated training
Ali et al. (2024a)	Explainable AI framework for autonomous vehicles	DoS attack detection with interpretable ML models	Detection accuracy and interpretability analysis in vehicular IoT environments
Qadir and Hashmy (2024)	Blockchain with multi-level verification	Smart Contracts and Robust Hashing	Validation efficiency and performance
El-Hajj et al. (2024)	Digital Twin + IDS	IDS + Resource Analysis	Attack detection accuracy, CPU utilization
Saheed et al. (2025)	CPS-IoT-PPDNN (Explainable DNN)	SHAP + Anomaly Detection	Accuracy (99.98 %), F1-score, SHAP

## 3 Materials and methods

### 3.1 Design of the integrated cyber-physical security architecture

The proposed architecture is designed as a modular and hierarchical solution for protecting distributed IoT infrastructures, where heterogeneous devices, insecure communication channels, and resource-constrained nodes create critical security challenges. The design is structured into three functional planes—physical, control, and security—that decouple data capture, orchestration, and active policy execution (Khalaf et al., 2024). This segmentation improves system performance in real-world deployments and supports the integration of advanced detection mechanisms and automated responses.

The physical plane includes sensors, actuators, and capture devices that interact directly with the environment. Temperature, humidity, proximity, and pressure sensors connect through low-power wireless links (e.g., ZigBee or Wi-Fi 802.11n) to an intermediate gateway. Actuators such as programmable relays, PWM controllers, and IP cameras are also managed by this gateway, which functions as an edge node for preprocessing and forwarding. Communication uses MQTT and CoAP (Pawar et al., 2023; Chen et al., 2024), selected for their compatibility with high-latency or low-bandwidth networks. MQTT follows a publish/subscribe model with configurable quality of service (QoS) levels, while CoAP operates over UDP, enabling REST-style queries with small payloads suitable for constrained sensors. All messages are encrypted with AES-256 in CBC mode and encapsulated with unique node identifiers to ensure origin authentication and traceability.

The control plane implements the logical layer, built on middleware that coordinates device management, event routing, and secure data access for internal and external services. This middleware adopts a microservices architecture deployed in Docker containers on edge nodes. The Event Broker, based on Mosquitto MQTT, distributes device events and applies ACLs with TLS mutual authentication to secure channels (Shin and Jeon, 2024). The Device Manager registers and monitors each device, using X.509 certificates for identity verification and periodic heartbeat updates to ensure availability. The API Gateway exposes RESTful endpoints secured with JWT tokens, providing the primary interface for monitoring, analysis, and remote management. All requests are encrypted with HTTPS over TLS 1.3, with forward secrecy and protected headers. These modules communicate over an encrypted and authenticated internal network, isolated from the physical plane and external access by dynamically updated firewalls. The middleware also normalizes input data, converting it to validated JSON structures with strict schemas and contextual classifications.

The security plane integrates mechanisms for detection, traffic analysis, and response execution. It consists of two main modules. The *anomaly detection engine*, implemented in TensorFlow Lite, performs real-time inference of suspicious device behaviors. Its design leverages heterogeneous features extracted from traffic statistics, middleware logs, and performance metrics, which include payload size, inter-arrival times, entropy scores, and authentication error rates. Supervised models, such as Random Forest and Decision Trees, were selected for their robustness with labeled data, while unsupervised approaches, such as autoencoders and DBSCAN clustering, enable the discovery of novel or previously unseen anomalies (Jony and Arnob, 2024). This hybrid combination ensures high detection sensitivity and resilience across different traffic conditions. The second module, the *encryption and active response unit*, enforces advanced cryptographic protection using AES-256-GCM and RSA-2048 (Luc et al., 2023). It integrates a rules engine capable of isolating nodes, regenerating keys, modifying communication paths, or blocking flows when risk conditions are detected. All actions are logged through an ELK Stack audit system, with JSON-formatted records digitally signed and stored on LUKS-encrypted volumes in read-only mode after each session.

An essential aspect of this architecture is that each module, including the Event Broker, Device Manager, API Gateway, anomaly detection engine, and response unit, is deployed as an autonomous microservice. This microservice-based decomposition ensures functional isolation, independent lifecycle management, and selective scalability of security-critical modules. For instance, the anomaly detection engine can be horizontally scaled under attack conditions without affecting authentication services. At the same time, the Event Broker can be optimized independently for throughput without interfering with auditing functions. This separation provides fault tolerance, facilitates updates without global downtime, and supports reproducible evaluation of each component in both physical and simulated environments.

**Figure 1:**
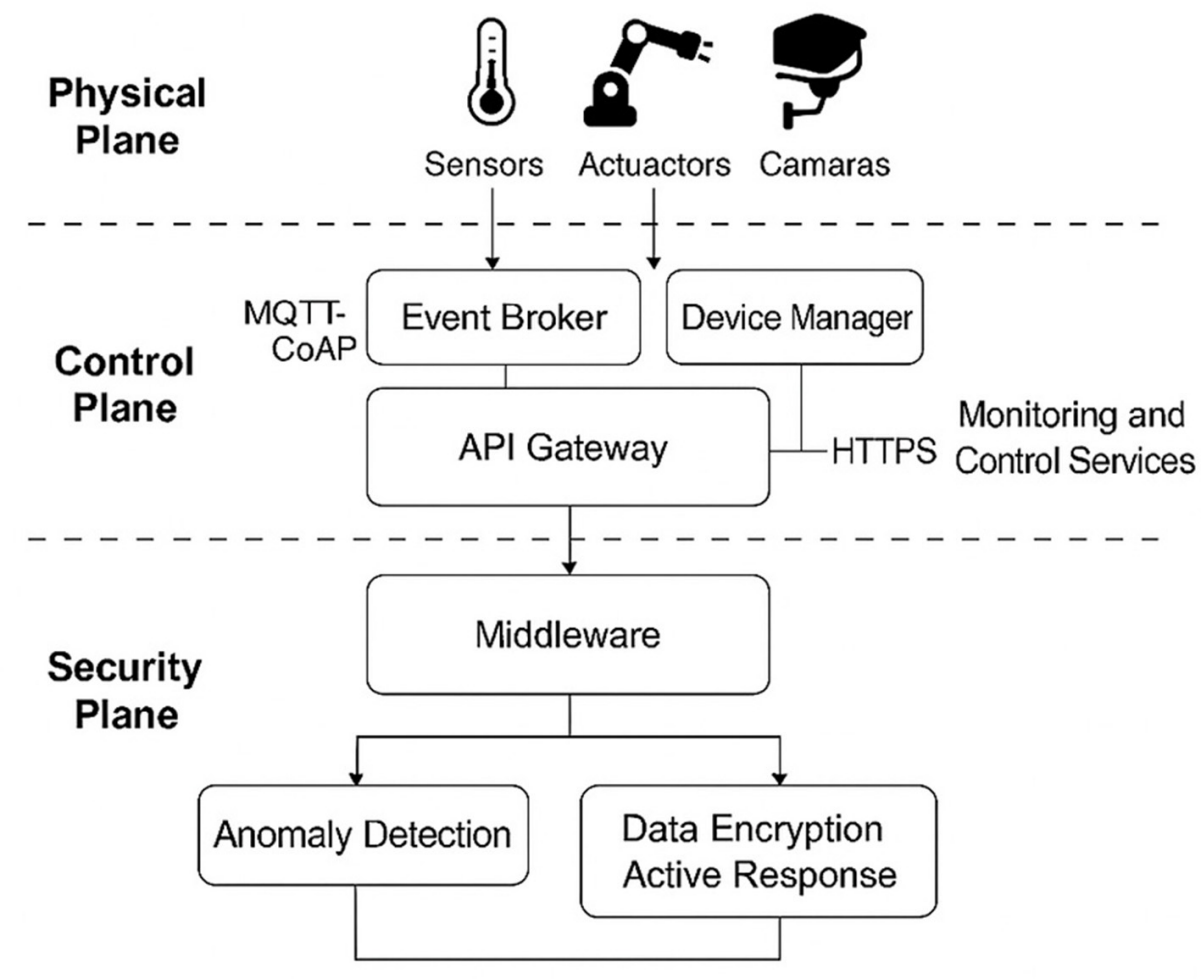
Integrated cyber-physical security architecture based on multi-layer middleware for IoT.

Figure 1 illustrates the integrated architecture, showing the relationships between middleware modules, secure communication paths, and the functional location of defense and monitoring mechanisms. Its decoupled design, with natively integrated detection and response, enables robust operation even under adverse network conditions or targeted attacks.

### 3.2 Multi-tier middleware implementation

#### 3.2.1 Functional middleware architecture

The middleware is implemented as a modular, distributed service-oriented architecture that orchestrates critical functions, including authentication, encryption, auditing, device management, anomaly detection, and access control. Each function operates as an autonomous microservice, communicating over an internal network secured with TLS and certificate-based authentication managed by a private certificate authority (local CA) (Wu et al., 2023).

The Event Broker, based on Mosquitto MQTT, acts as an asynchronous receiver node that routes device events to internal services. Its design supports logical flow segmentation through hierarchical topics, enabling priority assignment, channel-specific auditing, and access control via ACLs associated with each consumer microservice. The Device Manager maintains a dynamic registry of devices with unique identifiers, operational status, trust policies, and public keys. It incorporates heartbeat routines for availability verification and logging mechanisms protected by cryptographic challenges at the communication link. To address MQTT's inherent limitations under heavy load, the broker was configured with adaptive QoS levels (QoS 1 for regular traffic and QoS 2 for critical security messages), persistent sessions, and bounded message queues. In addition, dynamic firewall rules and broker-level rate limiting were implemented to mitigate flooding and DoS attempts, ensuring that malicious traffic is throttled without affecting high-priority events. This configuration was validated in controlled flooding scenarios, where the broker maintained stable throughput and bounded latency even under adversarial load. MQTT was selected as the backbone protocol because its lightweight design minimizes overhead in resource-constrained IoT environments. At the same time, the middleware's microservice decomposition compensates for scalability limitations through modular replication of brokers and separation of security-critical services. This provides a balanced trade-off between efficiency and resilience compared to heavier protocols such as AMQP or Kafka.

The Authentication Module validates every incoming request to the middleware, whether from monitoring services, physical nodes, or administrative users. Requests include JWT tokens signed with RSA-2048 keys and validated with timestamps to ensure authenticity and temporal validity. The Encryption Module applies AES-256 in GCM mode for data confidentiality in transit and at rest, complemented by ECDSA-P256 signatures for sensitive operations such as actuator control.

The Audit Layer consolidates structured logs generated by all modules and streams them to an Elasticsearch-Logstash-Kibana (ELK) pipeline (Yudhistira and Fitrisia, 2023). Events are indexed, signed, and stored in encrypted volumes managed by LUKS. Each record includes the origin, operation type, verification hashes, transaction results, and system actions. These records are immutable and externally auditable through verifiable signatures.

The Anomaly Detection Module integrates a lightweight inference engine built on TensorFlow Lite. It processes feature vectors extracted from device behavior in real time, including event emission rate, payload size, connection frequency, and deviations from historical patterns. The engine classifies traffic as usual or suspicious and triggers predefined response policies when a confirmed threat is detected.

**Figure 2:**
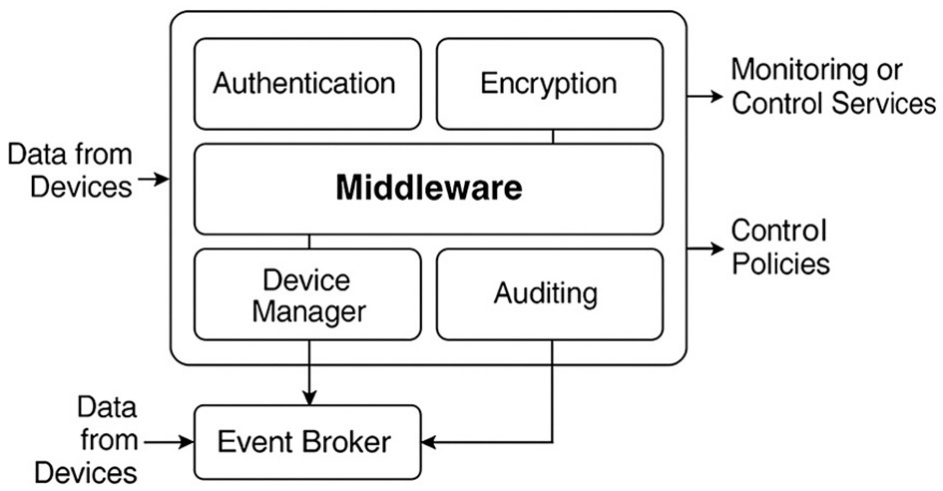
Internal functional structure of multi-layer middleware for IoT.

Figure 2 shows the interaction between these modules, illustrating the functional flow from event ingestion to processing and output toward monitoring services and external control mechanisms.

#### 3.2.2 Interoperability and abstraction of heterogeneous devices

A significant challenge in IoT environments is achieving interoperability among heterogeneous devices that differ in communication protocols, computational capacity, and manufacturing origin. To address this challenge, the middleware incorporates an abstraction layer based on logical objects called *IoTNodes*. Each IoTNode encapsulates the essential attributes of its corresponding physical device, including type (sensor or actuator), trust level, functional class, manufacturer, communication protocol, and configuration parameters.

A protocol-specific adapter manages every IoTNode. Adapters have been developed for MQTT, CoAP, HTTP/REST, and Bluetooth Low Energy (BLE). These adapters transform device requests into a standardized JSON data model (Robinson Joel et al., 2023). Acting as wrappers, they isolate device-specific logic from the middleware core, enabling all modules to operate on homogeneous data structures regardless of the underlying protocol.

At the network edge, multiprotocol gateways provide bidirectional translation between devices and middleware. For instance, a CoAP-based sensor can transmit packets to a gateway, which restructures them and forwards them via MQTT to the Event Broker without altering semantics or control. This hybrid strategy ensures compatibility with real-world environments where technological uniformity is rarely achievable.

#### 3.2.3 Technologies and tools used

The middleware was implemented using tools selected for their performance, community support, and compatibility with distributed environments. The logical core was developed in Python 3.10 with the Flask microframework, which provides lightweight REST routing, custom middleware handling, and session control. JWT-based authentication was integrated with the Flask-JWT-Extended library, while PyCryptoDome supported advanced cryptographic operations. Data serialization and validation were handled with Marshmallow, and endpoints were deployed using Gunicorn on Linux servers.

The messaging layer relies on Mosquitto MQTT, configured with TLS 1.3 mutual authentication, topic-based access control, and critical message retention to ensure consistency. Log and event processing were managed with Logstash, while Elasticsearch provided storage and indexing, and Kibana enabled visualization. Each middleware module is containerized with Docker and orchestrated using docker-compose.yml definitions, which specify virtual networks, persistent volumes, and encrypted environment variables (Malhotra et al., 2024).

Functional validation was conducted in laboratory environments on Ubuntu 22.04 LTS servers equipped with eight cores and 16 GB of RAM. Postman and JMeter were used for load testing, and Prometheus with Grafana supported internal monitoring and metric visualization.

#### 3.2.4 Implementation model: microservices vs. SOA

The adoption of a microservices-based architecture, instead of a traditional SOA, addresses the need to fully decouple functional modules and thereby improve scalability, fault tolerance, and maintainability. Conventional SOA relies on a central integration bus (Enterprise Service Bus), which introduces a single point of failure and complicates lifecycle management (Bonilla et al., 2024). In contrast, the proposed microservices model deploys each component –authentication, device management, encryption, auditing, and detection—as an autonomous unit with its own lifecycle, dependencies, and communication channel.

This design enables partial updates without global outages, the deployment of environment-specific versions (production, testing, or edge), and horizontal scaling of modules according to workload. For instance, authentication and analytics modules were dynamically scaled during denial-of-service attack simulations while maintaining overall system stability. In addition, microservices simplify integration with orchestration tools such as Docker Compose and allow seamless migration to platforms like Kubernetes when higher levels of automation are required in large-scale production environments (Decker et al., 2022).

### 3.3 Integrated security mechanisms

#### 3.3.1 Authentication and identity management model

Access control in the middleware follows a hierarchical authentication model that combines signed tokens with cryptographic device validation. Each IoT node attempting to join the system must present a public key registered in the Device Manager and sign a challenge message with its private key. After successful verification, the system issues a JSON Web Token (JWT) containing the node identifier, authorization profile, and an expiration time in UNIX format. The token is signed with the RSASSA-PSS algorithm using RSA-2048 keys, in compliance with the RFC 7519 standard (Rana et al., 2023). Token validation occurs at the gateway of each microservice, ensuring mutual authentication and fine-grained permission control.

For external services accessing middleware endpoints (e.g., monitoring dashboards or remote control applications), a complementary OAuth 2.0 scheme is applied. A local authorization server validates client credentials, and the resulting tokens are subject to expiration, manual revocation, and on-demand renewal.

#### 3.3.2 Cryptographic protection and key management

The system enforces encryption in transit and at rest to guarantee data confidentiality and integrity. All communication between devices, gateways, and middleware modules is secured with TLS 1.3, using ECDHE for ephemeral key exchange to enable forward secrecy. Data stored on disk, including audit logs, critical events, and node metadata, is encrypted with AES-256 in GCM mode, which ensures authenticity through embedded message authentication codes (MACs).

Key management is handled locally through a configurable Key Management System (KMS) that applies rotating policies. Each symmetric key has a predefined lifetime (e.g., 48 hours) or an operation-based threshold. Once the limit is reached, the system generates a new key, encrypts the previous one with a backup public key, and retains it for a four-hour grace period. This mechanism supports validation of pending transactions and delayed synchronization across distributed components. Keys are stored only in volatile memory and are loaded on demand to minimize persistent exposure.

#### 3.3.3 Anomaly detection engine

The anomaly detection engine constitutes the core of the active security mechanisms. It is implemented on TensorFlow Lite and optimized for execution on edge nodes. Operating in real time, the engine processes events from the Event Broker. Each event is preprocessed into a feature vector $\vec{x}\in {\mathbb{R}}^{n}$, which includes attributes such as message emission rate, accumulated latency, average payload size, deviation from historical behavior, and the number of recent disconnections.

The detection pipeline integrates multiple classifiers (Random Forest, Decision Tree, and a compact Autoencoder), but the final decision layer is implemented as a lightweight neural unit in TensorFlow Lite. Let *f*:ℝ^*n*^ → [0, 1] denote this probabilistic decision function, defined as:


$$\begin{array}{l} S\left(\vec{x}\right)=f\left(\vec{x}\right)=\sigma \left(W\vec{x}+b\right), \end{array}$$ (1)

where *W* is the weight matrix fitted during supervised training and σ(·) is the activation function (sigmoid). The output $S\left(\vec{x}\right)$ represents the anomaly score produced by the final decision layer, aggregating the predictions of the ensemble. A device is classified as potentially at risk if:


$$\begin{array}{l} S\left(\vec{x}\right)>\theta , \end{array}$$ (2)

where θ is an adaptive threshold determined by statistical analysis on the validation set.

The anomaly detection engine is based on a compact Multilayer Perceptron (MLP) implemented in TensorFlow Lite, designed to combine nonlinear learning capacity with the computational efficiency required for edge deployment. This architecture was selected as an intermediate approach between a purely linear logistic regression and a deep neural network, ensuring sufficient expressiveness for pattern recognition while maintaining sub-100 ms inference latency on constrained devices. The probabilistic function $f\left(\vec{x}\right)$ described in Equation 1 corresponds to the output layer of this MLP, trained to aggregate the predictions of the ensemble classifiers. The model comprises an input layer of 26 neurons (one per normalized feature), a hidden layer with 16 ReLU-activated units, and a single sigmoid output neuron that produces the anomaly probability $S\left(\vec{x}\right)\in \left[0,1\right]$. It is trained in TensorFlow using binary cross-entropy as the loss function and the Adam optimizer with a learning rate of 10^−3^. Training followed a supervised scheme with early stopping (patience = 10 epochs) and a batch size of 128, ensuring convergence without overfitting. The adaptive decision threshold θ in Equation 2 was derived empirically as θ = μ_*S*_+1.5σ_*S*_, where μ_*S*_ and σ_*S*_ denote the mean and standard deviation of $S\left(\vec{x}\right)$ on the validation set. This formulation maintains sensitivity under class imbalance while minimizing false positives. The anomaly score $S\left(\vec{x}\right)$ thus represents the probability that an observed event deviates significantly from the learned baseline. Scores near 1 indicate critical anomalies requiring immediate containment, while values below θ correspond to nominal behavior incorporated into the adaptive historical profile.

To address the reviewer's concern regarding the integration of the auxiliary classifiers within the final anomaly detection process, we explicitly clarify that the Random Forest, Decision Tree, and Autoencoder models do not operate as standalone decision mechanisms. Instead, each model produces a complementary anomaly indicator *s*_RF_, *s*_DT_, and *s*_AE_, respectively–derived from supervised probabilities (RF and DT) and reconstruction error (AE). These outputs are concatenated with the normalized behavioral feature vector $\vec{x}$ to construct a 29-dimensional meta-input vector


$$\begin{array}{l} \vec{z}=\left[x_{1},\ldots ,x_{26},s_{\text{RF}},s_{\text{DT}},s_{\text{AE}}\right], \end{array}$$ (3)

**Figure 3:**
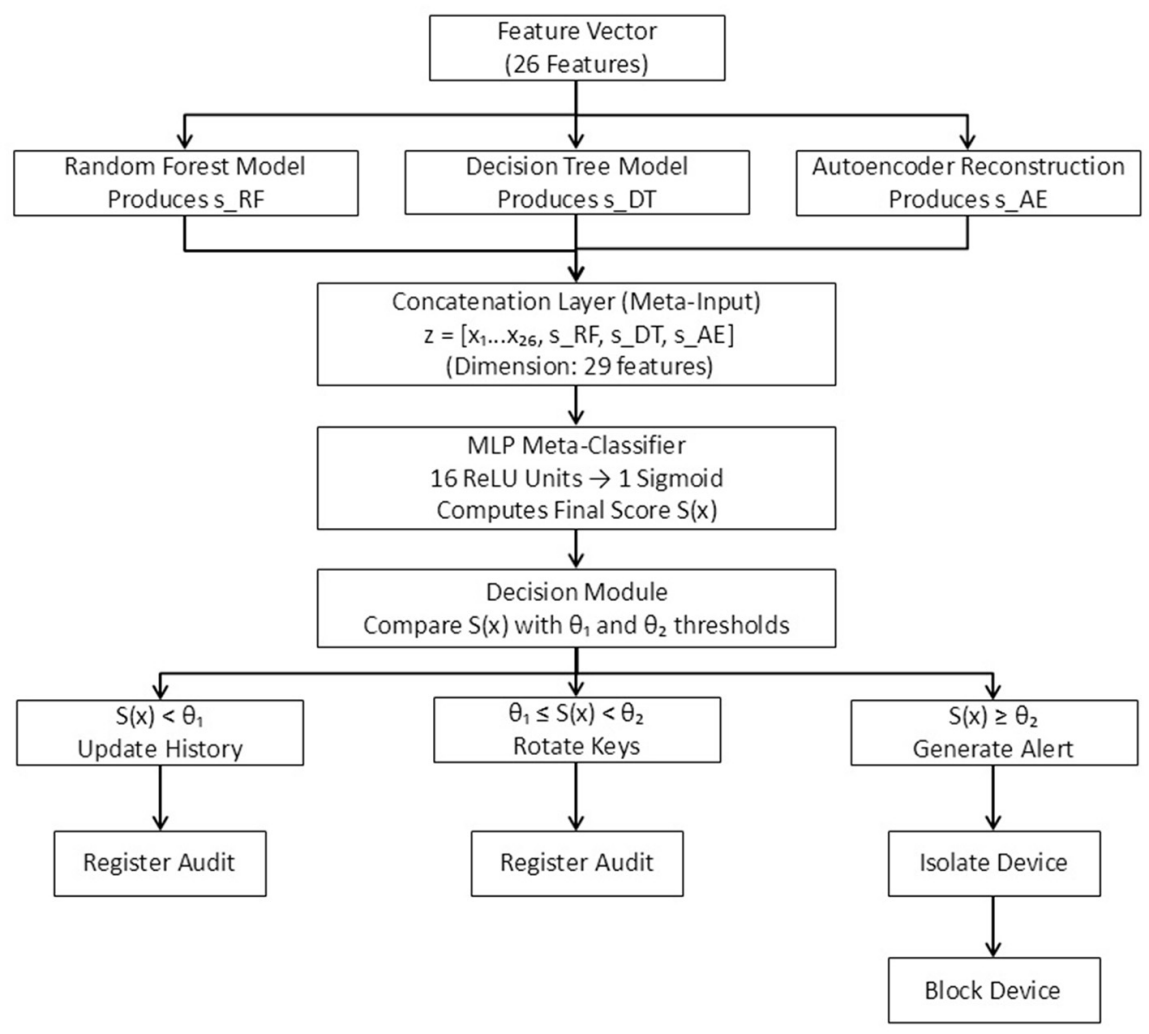
Complete anomaly detection pipeline. The auxiliary models (RF, DT, AE) generate complementary anomaly indicators that are concatenated with the feature vector and processed by an MLP meta-classifier. The final anomaly score S(x) is evaluated using a dual-threshold decision module that triggers automated mitigation actions.

The lightweight MLP described above processes $\vec{z}$, acting as a meta-classifier that aggregates heterogeneous anomaly cues and computes the final anomaly score $S\left(\vec{x}\right)$. This stacked-generalization scheme enables nonlinear fusion of both raw device behavior and auxiliary detection signals, while maintaining real-time inference efficiency on edge nodes. Figure 3 illustrates the full detection pipeline, showing the flow from feature extraction to ensemble signal generation, meta-classification, and threshold-based autonomous response.

**Figure 4:**
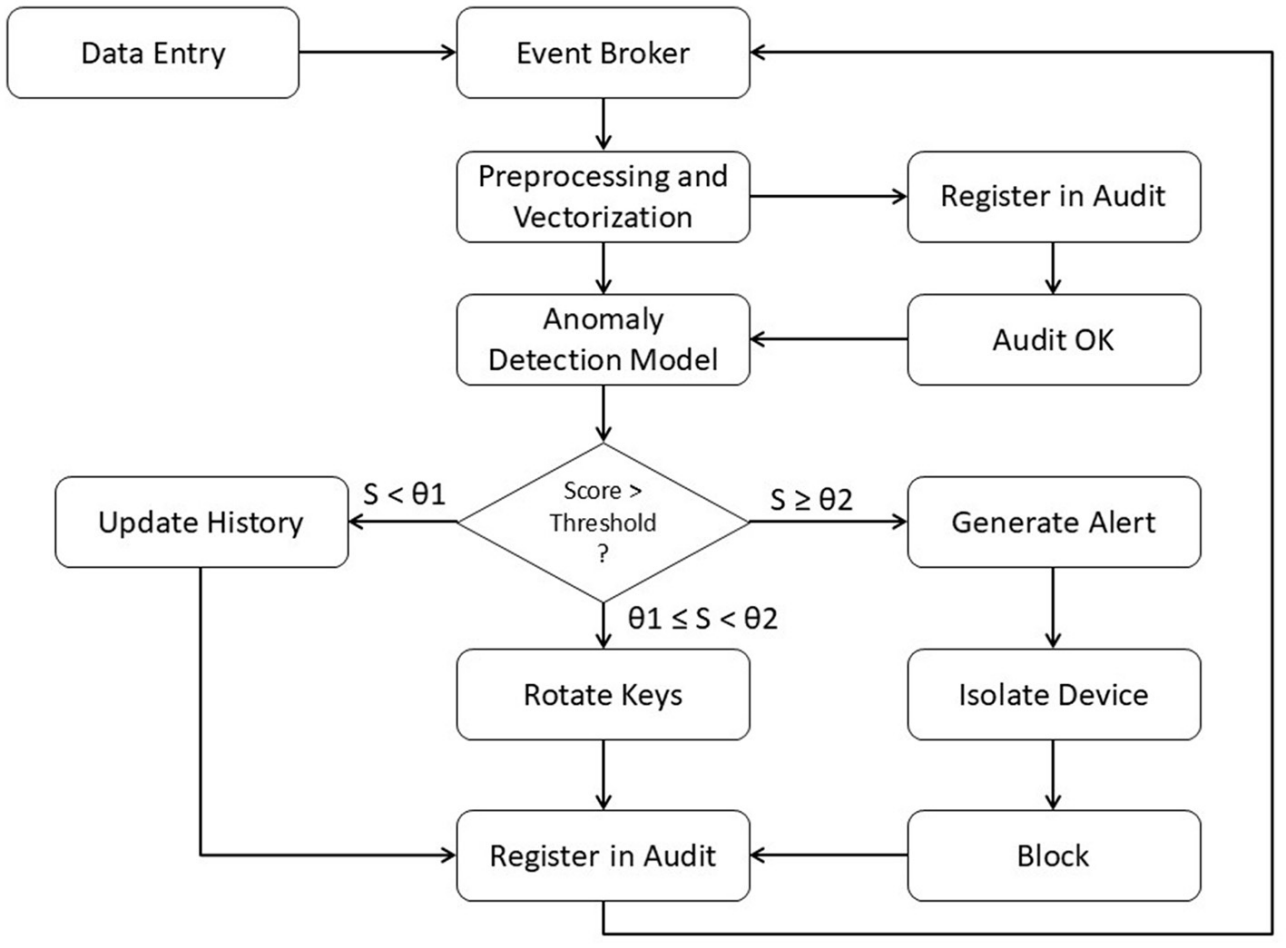
Decision flow for anomaly detection and autonomous response in middleware.

When this condition is evaluated, the engine triggers the automatic response flow illustrated in Figure 4. The flow models the complete cycle from event reception to the activation of security policies, with decisions defined by two adaptive thresholds (θ_1_ and θ_2_). Events with scores below θ_1_ are considered low risk and are incorporated into the historical baseline through the *Update History* process. Events with scores between θ_1_ and θ_2_ activate preventive measures, such as cryptographic key rotation, while all activity is recorded in the audit log. For events with scores above θ_2_, the system generates an alert and executes strict containment policies, including device isolation and flow blocking, which are also registered in the audit system.

#### 3.3.4 Automated response system

The automated response system enforces containment policies whenever an event exceeds the predefined risk threshold. These policies are encoded as rules in a decision engine implemented with Drools and are composed of conditions defined over input attributes combined with logical operators. The pseudocode of the evaluation and response process is shown in Algorithm 1.

Algorithm 1 Anomaly Detection and Autonomous Response Procedure

**Require:** Incoming event *e*
**Ensure:** Execution of security response if anomaly is detected
1: *x* ← EXTRACTFEATUREVECTOR(*e*)
2: *score* ← ANOMALYMODEL(*x*)
3: LogAudit(*e, score*)
4: **if** *score* > *threshold* **then**
5:  GenerateAlert(*e*.*source*)
6:  **if** *e*.*type* = = “actuator” **then**
7:   IsolateDevice(*e*.*source*)
8:   RevokeCredentials(*e*.*source*)
9:  **else**
10:   ReconfigureChannel(*e*.*source*)
11:  **end if**
12: **else**
13:  UpdateHistory(*e*.*source, score*)
14: **end if**

This procedure is executed concurrently for each incoming event. If the affected device is an actuator, the system prioritizes immediate isolation and credential revocation to prevent malicious commands. For sensors, the response is more conservative, involving channel reconfiguration or continuous monitoring of their behavior.

### 3.4 Hybrid evaluation environment

The proposed system was validated using a hybrid approach that combines testing in a physical environment with controlled simulations of attack scenarios, legitimate traffic, and edge conditions. This strategy enables the assessment of middleware performance, detection accuracy, resilience, and response efficiency against both known and emerging threats. By integrating real and simulated conditions, the evaluation reproduces behaviors that are difficult to capture in isolated settings, ensuring a more comprehensive and realistic system assessment.

#### 3.4.1 Physical experimental environment

The physical environment was deployed on a private local area network (LAN) of heterogeneous nodes interconnected via a Gigabit Ethernet switch and a 2.4 GHz Wi-Fi network. The central edge device was a Raspberry Pi 4 Model B with 4 GB RAM and Raspberry Pi OS 64-bit, configured as a gateway, edge node, and middleware integration agent.

Multiple endpoint devices were connected, including DHT22 sensors (temperature and humidity), PIR sensors (motion detection), ESP32-CAM IP cameras, and AC load control modules. These devices communicated via MQTT and HTTP protocols and were configured to emit events at intervals between 5 and 60 s, emulating heterogeneous operational patterns. The middleware was deployed directly on the Raspberry Pi through Docker, with each module running in an isolated container. The local network was logically segmented into virtual subnets managed by the UFW firewall, enabling the emulation of restricted communication conditions and semi-trusted environments. External communication was limited to remote monitoring panels secured with encrypted SSH and HTTPS tunnels.

System monitoring during tests employed Wireshark and Netdata to track network traffic, node latency, CPU utilization, and system behavior under both normal and load conditions. Experiments were repeated over 24-h cycles, generating more than 180,000 logged events. These logs were used to evaluate system performance and to refine the anomaly detection model. This setup is consistent with prior work, such as Plazas Olaya et al. (2024), who validated a microservices-based IDS with real IoT traffic, reporting F1-scores above 98 % and accuracies near 99 % in detection tasks.

#### 3.4.2 Simulated environment

The simulated environment was implemented using two complementary platforms: NS-3 for network and traffic simulation, and Contiki Cooja for low-power IoT device emulation. Both tools support protocol-level modeling, event replay under adverse conditions, and targeted attack simulation with fine-grained control.

In NS-3, a network of 50 IoT nodes was configured with a mixed topology (mesh and star) to represent heterogeneous links in terms of latency, packet loss, and jitter. Nodes emulated virtual sensors, cameras, and actuators, generating traffic over MQTT or UDP. The simulation included baseline traffic, periodic bursts, unexpected events, and noisy transmissions.

In Contiki Cooja, Zolertia Z1 nodes were deployed with full-stack simulation (from the MAC to the application layer) to evaluate detection performance under highly volatile network conditions. Nodes generated events every 10 s, while scheduled faults such as intermittent disconnections, channel hopping, and payload modification were introduced to reproduce configuration errors and anomalous behaviors.

Both environments were synchronized with the real middleware deployed on a host machine through a bridge interface that redirected simulated traffic to the physical Event Broker. This setup enabled analysis of how the middleware responded to synthetic events with known patterns, leveraging reproducible boundary conditions for validation.

#### 3.4.3 Simulated attack scenarios and normal conditions

The evaluation of the detection and response system focused on three attack categories frequently observed in IoT environments:

- Identity spoofing: Nodes were simulated to replicate the identity of valid devices using modified packets. The system detected anomalies through traffic pattern changes, inconsistent metadata, and abrupt increases in sending rates, which raised anomaly scores above the threshold.
- Event flooding: Devices were configured to emit events at a rate ten times higher than expected (every 0.5 s). This produced bottlenecks in the Event Broker and validated the effectiveness of automatic isolation modules and key rotation mechanisms in controlling saturation.
- DoS: External nodes executed Hping3 and slowloris attacks, generating traffic patterns consistent with resource exhaustion. The system responded by blocking the malicious sources once the risk threshold was exceeded and logged all incidents in the structured audit trail.

In addition, periods of regular operation were simulated with balanced traffic, stable device performance, and minimal error rates. These normal conditions were critical for estimating false-positive rates and refining the detection threshold through cross-validation. Consistent with the methodology of (El-Hajj et al., 2024), who employed a digital twin to simulate Hping3 and NMAP attacks, scenarios were repeated multiple times to confirm model stability and reproducibility.

### 3.5 Dataset and input data

The implementation of anomaly detection and automated response mechanisms requires a solid foundation of structured data that captures both legitimate behavior and attack patterns in IoT networks. To this end, a composite dataset was constructed from heterogeneous sources, combining logs generated in the physical experimental environment, synthetic data from the simulated environment, and publicly available datasets widely used in cyber-physical security research.

#### 3.5.1 Data sources used

Three publicly available datasets were selected for their contextual richness, granular labeling, and relevance to modern IoT scenarios:

- Edge-IIoTset (Ferrag et al., 2022): A large-scale IIoT/IoT dataset generated on a multi-layer testbed. It contains *over 72 million records*; the raw PCAP portion is *69.3 GB* and the extracted flow CSV is *16.7 GB*. The release includes rich metadata suitable for ML-based IDS (dozens of raw features later reduced via feature selection).
- IoT-23 (Garcia et al., 2020): A labeled dataset with 23 capture scenarios (20 malicious and 3 benign) collected by the Stratosphere Laboratory (CTU, Prague). It provides original .pcap and Zeek labeled flow files per scenario. The official Zenodo bundle (iot_23_datasets_full.tar.gz) is 21.5 GB; scenarios were captured in 2018–2019 and released in 2020.
- CIC IoT 2023 (Canadian Institute for Cybersecurity, 2023): A real-time IoT attack dataset comprising *105 devices* and *33 attacks* grouped into *seven* categories (DDoS, DoS, Recon, Web-based, Brute Force, Spoofing, Mirai), with benign traffic and multiple export formats for downstream ML workflows.

All datasets were obtained in PCAP format, processed, and validated using specialized tools such as pandas and Wireshark, as well as custom validation scripts. They were subsequently integrated with middleware-generated data, including Event Broker and Device Manager logs, internal audit records, and CPU/RAM usage metrics collected through Prometheus.

#### 3.5.2 Data types and their integration

The data was grouped into three categories: network traffic, middleware logs, and performance metrics. Network traffic corresponds to packets captured between devices and middleware modules, including attributes such as ports, payload size, inter-arrival times, TCP/UDP flags and flow duration. This traffic was processed with tshark and normalized into logical flows (unidirectional or bidirectional).

The middleware generated Access and audit logs to record authentications, critical events, policy changes, and automated responses. These entries were processed in real time via Logstash and indexed in Elasticsearch. Performance metrics, collected with node_exporter, include CPU, memory, and activity usage per module. These values provide insight into overload conditions and atypical behavior patterns.

Each record was transformed into a normalized numerical feature vector suitable for the detection models. Categorical fields (e.g., device type, protocol, traffic source) were converted with one-hot encoding, while numerical attributes were scaled using min-max normalization:


$$\begin{array}{l} x^{\prime }=\frac{x-x_{\text{min}}}{x_{\text{max}}-x_{\text{min}}}, \end{array}$$ (4)

where *x* is the original attribute value, and *x*_min_, *x*_max_ are the minimum and maximum values observed in the training set. This guarantees that each dimension of the vector $\vec{x}\in {\mathbb{R}}^{n}$ is bounded in [0, 1], improving numerical stability and convergence of machine learning models.

#### 3.5.3 Feature representation and encoding

The final feature vector integrated network, middleware, and performance-level attributes, all derived from the unified dataset described previously. Each input sample was represented as a normalized vector $\vec{x}\in {\mathbb{R}}^{26}$, where the 26 dimensions correspond to the most discriminative variables identified during the feature selection stage. These attributes capture behavioral patterns associated with message flow dynamics, resource utilization, and protocol activity across devices and services.

The feature composition included continuous variables such as message emission rate, latency, payload size, and CPU or memory consumption; discrete variables derived from temporal counters, such as the number of authentication failures or reconnection attempts; and categorical variables that describe contextual states, including communication protocol, device role, or policy type. All categorical features were transformed using one-hot encoding to ensure numerical compatibility with the learning models. At the same time, continuous and discrete attributes were scaled to the [0, 1] interval through min-max normalization, as defined in Equation 4. This normalization ensured that all features contributed proportionally to the model's convergence and avoided dominance of attributes with larger magnitudes.

Before normalization, time-dependent variables were aggregated within non-overlapping windows of five seconds to reduce the effect of transient spikes caused by message bursts or queue accumulation in the broker. Statistical smoothing operations (mean and standard deviation) were applied within each window, enabling the model to capture stable behavioral tendencies rather than instantaneous fluctuations. Each vector $\vec{x}$ thus encodes both instantaneous metrics and short-term temporal dependencies representative of device or service performance under dynamic load.

After preprocessing, the resulting feature matrix preserved a consistent dimensionality and balanced distribution between classes, facilitating uniform training across all evaluated models. The final dataset retained 26 features per record, all expressed as real-valued components within a standard normalized scale, ensuring that subsequent inference in TensorFlow Lite could be efficiently executed on edge nodes with minimal computational overhead.

#### 3.5.4 Cleaning and preprocessing process

Before training, several data-cleaning techniques were applied to ensure the quality. Of the data, Incomplete records and duplicate entries were removed, and those with inconsistencies between packet size and declared protocol were filtered. Missing values in CPU-usage fields and timestamps were interpolated using a second-order polynomial fitting method.

Subsequently, a feature vector $\vec{x}$ was constructed for each event:


$$\begin{array}{l} \vec{x}=\left[f_{1},f_{2},f_{3},\ldots ,f_{n}\right], \end{array}$$ (5)

where each *f*_*i*_ represents a selected metric, for example:

- *f*_1_: device event rate per second
- *f*_2_: average payload size
- *f*_3_: round-trip latency (RTT)
- *f*_4_: number of authentication failures in the last 10 minutes
- *f*_5_: average CPU usage of the sending module
- *f*_*n*_: semantic similarity score with respect to previous events

In the final configuration, each feature vector had dimension *n* = 26. Following feature-importance analysis, the original set of 58 candidate attributes was reduced to the 26 most discriminative variables using the Gini index and information-gain criteria.

The resulting vectors were labelled according to the data source (legitimate or anomalous) and split into training (70 %), validation (15 %), and test (15 %) sets using StratifiedShuffleSplit to preserve class balance.

After cleaning and integration, the unified dataset comprised approximately 620,000 labeled records, of which 53% were benign and 47% anomalous. Approximately 40% of records originated from Edge-IIoTset, 35% from IoT-23, 20% from CIC IoT 2023, and 5% from internal middleware logs.

#### 3.5.5 Hyperparameter selection and model justification

The anomaly detection models were configured with hyperparameters optimized through stratified 5-fold cross-validation to ensure robustness and reproducibility. For the Random Forest classifier, the number of trees was set to *n*_*estimators* = 200, with maximum depth = 20 and minimum samples per split = 4, parameters selected to balance variance reduction and computational efficiency. The Decision Tree baseline used Gini impurity as the splitting criterion with maximum depth = 15, preventing overfitting while maintaining interpretability.

For the autoencoder, the latent space dimension was fixed at 16, with ReLU activation in the encoding layers and sigmoid in the reconstruction layer. The model was trained with Adam optimizer (α = 0.001, β_1 = 0.9, β_2 = 0.999) for 100 epochs, batch size = 128, and early stopping with patience = 10 to avoid unnecessary computation. DBSCAN clustering employed ε = 0.5 and minimum samples = 10, empirically determined through silhouette analysis on the validation set.

These hyperparameters were selected after grid search and empirical sensitivity analysis, considering both classification accuracy and computational constraints of edge nodes. This procedure ensured that the models retained high detection performance (F1-scores >0.93) while remaining feasible for real-time deployment in resource-constrained IoT environments.

### 3.6 Evaluation metrics and experimental procedure

System validation was structured along two axes: the performance of the anomaly detection engine on labeled events, and the efficiency of the middleware under different load conditions. Quantitative metrics were selected based on specialized literature and established practices for IoT security evaluation (Saheed et al., 2025).

#### 3.6.1 Detection metrics

The detection system was assessed using both standard classification metrics and indicators designed to handle class imbalance and asymmetric errors. In particular, accuracy and F1-Score were complemented with the Matthews Correlation Coefficient (MCC) and Cohen's Kappa index. Let the confusion matrix be defined as:

- True positives (TP)
- False positives (FP)
- True negatives (TN)
- False negatives (FN)

The key metrics are defined as follows:

Weighted F1-Score:


$$\begin{array}{l} F_{1}=2\cdot \frac{\text{Precision}\cdot \text{Recall}}{\text{Precision}+\text{Recall}}=\frac{2TP}{2TP+FP+FN}, \end{array}$$ (6)

where *Precision* = *TP*/(*TP*+*FP*) and *Recall* = *TP*/(*TP*+*FN*).

Matthews Correlation Coefficient (MCC):


$$\begin{array}{l} \text{MCC}=\frac{\left(TP\cdot TN-FP\cdot FN\right)}{\sqrt{\left(TP+FP\right)\left(TP+FN\right)\left(TN+FP\right)\left(TN+FN\right)}}, \end{array}$$ (7)

which provides a balanced evaluation even under strong class imbalance, with values ranging from −1 (inverse prediction) to +1 (perfect prediction).

Cohen's Kappa (κ):


$$\begin{array}{l} \kappa =\frac{p_{o}-p_{e}}{1-p_{e}}, \end{array}$$ (8)

where *p*_*o*_ is the proportion of observed agreement and *p*_*e*_ is the expected agreement by chance. This metric is relevant when evaluating systems that process events from heterogeneous sources with dominant marginal classes.

Finally, the Area Under the ROC Curve (AUC-ROC) quantifies the model's discrimination capability, representing its ability to distinguish between legitimate and anomalous events across different decision thresholds.

#### 3.6.2 System performance metrics

The operational efficiency of the middleware was assessed in terms of latency, resource utilization, throughput under load, and sensitivity to anomalous events in real time. The main metrics are defined as follows:

Average Event Processing Latency (*L*):


$$\begin{array}{l} L=\frac{1}{n}\overset{n}{\underset{i=1}{\sum }}\left(t_{i}^{\text{output}}-t_{i}^{\text{input}}\right), \end{array}$$ (9)

where $t_{i}^{\text{input}}$ and $t_{i}^{\text{output}}$ denote the arrival and completion times of event *i*.

Average CPU and RAM Consumption per Module (*U*_cpu_, *U*_ram_): Resource usage was monitored with Prometheus. The normalized relative load for resource *k* (CPU or RAM) at time *t* was defined as:


$$\begin{array}{l} U_{k}\left(t\right)=\frac{r_{k}\left(t\right)}{r_{k,\text{max}}}, \end{array}$$ (10)

where *r*_*k*_(*t*) is the observed consumption and *r*_*k*, max_ is the maximum permitted threshold.

Throughput (τ):


$$\begin{array}{l} \tau =\frac{N}{T}, \end{array}$$ (11)

where *N* is the number of events successfully processed in a time interval *T*, throughput was measured under normal load and attack conditions to evaluate potential degradation.

Traffic Entropy (*H*): Entropy was used as an indicator of dispersion and irregularity in observed flows:


$$\begin{array}{l} H=-\overset{n}{\underset{i=1}{\sum }}p_{i}\log_{2}\left(p_{i}\right), \end{array}$$ (12)

where *p*_*i*_ is the probability of occurrence of event *i*, sudden increases in entropy are associated with disorganized or synthetic traffic, characteristic of flooding or spoofing attacks.

#### 3.6.3 Experimental procedure

The evaluation was conducted in eight experimental scenarios combining legitimate traffic, attack patterns (spoofing, flooding, and DoS), and hybrid conditions with ambiguous events. Each scenario was executed five times in an isolated environment, with all services restarted between runs to eliminate residual state. Experiments were performed within 24-h windows, with a minimum runtime of 100 min per iteration.

The tools employed were:

- Prometheus and Grafana: for real-time monitoring of operational metrics.
- Scikit-learn and TensorFlow: for statistical inference and model evaluation.
- JMeter and custom MQTT publishers: for generating synthetic traffic and stress-testing the system.
- Wireshark and Netdata: for network analysis and operating system resource profiling.

All metrics were stored in structured logs, processed with pandas, and visualized with Matplotlib and Seaborn. Statistical hypothesis tests, including ANOVA and paired *t*-tests, were applied to assess the significance of performance differences across scenarios, with a 95 % confidence level (α = 0.05).

## 4 Results

### 4.1 Performance of the anomaly detection system

The detection system was evaluated in five operational scenarios: legitimate traffic, known attacks, hybrid attacks, unlabeled events, and background noise. Table 2 summarizes the average F1-Score, MCC, and AUC-ROC values obtained by the model in each case. These results allow analysis of the system's ability to distinguish between anomalous and legitimate traffic and to assess robustness under ambiguous or noisy conditions.

**Table 2:** Performance of the detection system under different scenarios.

**Scenario**	**F1-score**	**MCC**	**AUC-ROC**
Legitimate traffic	0.971	0.961	0.994
Known attacks	0.938	0.917	0.989
Hybrid attacks	0.894	0.831	0.963
Unlabeled events	0.763	0.702	0.912
Background noise	0.681	0.623	0.878

In the legitimate traffic scenario, the model achieved an F1-Score of 0.971 and an MCC of 0.961, reflecting high accuracy and balance between true positives and false negatives. The AUC-ROC value of 0.994 confirms the classifier's ability to clearly separate legitimate events from potentially anomalous ones across different thresholds.

For known attacks, including DoS, spoofing, and flooding, the system maintained an F1-Score of 0.938 and an MCC of 0.917, demonstrating high sensitivity to malicious events represented in the training datasets. The AUC of 0.989 further validates its strong discrimination capability. These results confirm the effectiveness of supervised training with CIC IoT 2023 and IoT-23, showing that the system generalizes well to common attack patterns.

Hybrid scenarios, where legitimate sequences were combined with anomalous behaviors, produced a progressive degradation in performance, reaching an F1-Score of 0.894 and an MCC of 0.831. This decline is expected, as hybrid events present ambiguous patterns not fully captured in training. The AUC-ROC of 0.963 reflects larger regions of uncertainty in the decision space. Nevertheless, the system maintained acceptable discrimination in these conditions. In the case of unlabeled events, such as traffic from new device types or unstructured formats, performance decreased further (F1-Score of 0.763, MCC of 0.702), reflecting the semantic and temporal divergence from the training space. Finally, background noise, generated by spurious or erratic data, led to the lowest results (F1-Score of 0.681, MCC of 0.623), indicating increased false positives.

**Figure 5:**
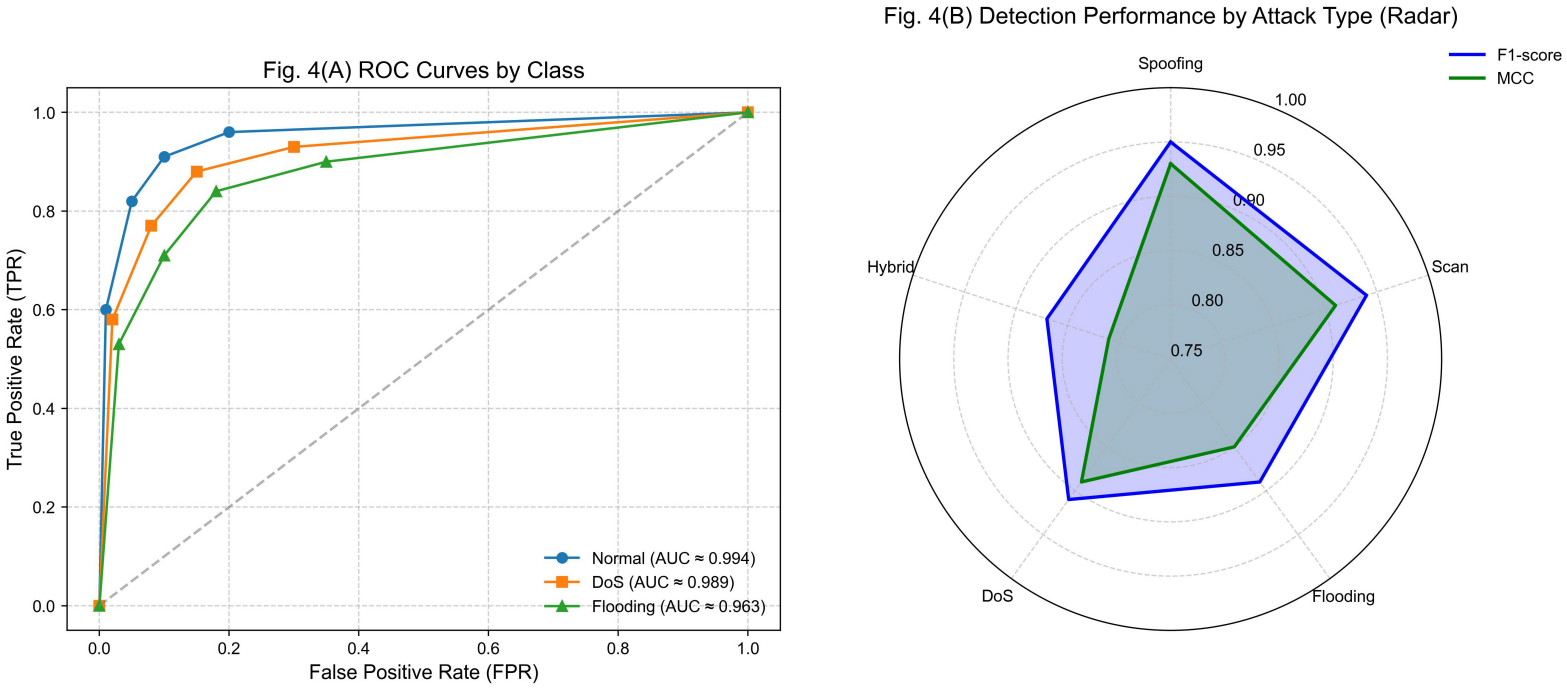
Performance evaluation of the detection system. **(A)** ROC curves for legitimate, DoS, and flooding classes. **(B)** Radar chart comparing F1-Score and MCC by attack type.

Figure 5 illustrates these findings. Figure 4A shows ROC curves for three representative classes: legitimate traffic, DoS, and flooding. For legitimate traffic, the ROC curve approaches the ideal point (0,1) with an AUC close to 0.994, confirming the system's ability to identify benign events without false alarms correctly. The curve remains steep for DoS attacks, with an AUC ≈0.989, highlighting effective detection of repetitive, high-load patterns. For flooding, the curve is flatter and slightly overlaps with ambiguous events, resulting in an AUC of 0.963, the lowest among the cases analyzed.

This behavior aligns with the hybrid simulations, where flooding was the most susceptible to confusion due to variability and similarity to legitimate traffic. The ROC curves illustrate how the inference engine retains high accuracy for structured attack patterns while showing reduced discrimination for intermittent or asynchronous events.

Figure 4B presents a radar chart comparing F1-Score and MCC across spoofing, scanning, flooding, DoS, and hybrid attacks. The strongest performance appears in spoofing and scanning, with F1-Scores of 0.95 and 0.94 and MCC values above 0.91. These high results are explained by the regularity of their patterns, such as invalid authentication sequences or consecutive port scans. In contrast, hybrid attacks exhibited the lowest performance (F1-Score of 0.87, MCC of 0.81), reflecting their structural complexity and statistical similarity to legitimate traffic. Flooding and DoS achieved intermediate scores, both above 0.89, but with higher variance across executions.

### 4.2 Label harmonization and class balance analysis

To ensure that the reported performance metrics were not biased by inconsistencies among heterogeneous datasets, a harmonization and balancing process was carried out before model training. Traffic categories from Edge-IIoTset, IoT-23, and CIC IoT 2023 were consolidated into six unified labels: *benign, DoS, scan, spoofing, exfiltration*, and *malware*. The mapping of equivalent attack types across datasets was validated through metadata inspection, protocol-level correlation, and alignment of ground-truth annotations to guarantee semantic consistency.

The initial distribution analysis revealed a substantial imbalance, with DoS and scan events dominating the combined dataset by up to five times compared to minority classes, such as exfiltration or spoofing. Balancing was achieved through a hybrid resampling scheme combining inverse-frequency weighting and synthetic minority oversampling (SMOTE) applied to the feature space. Overrepresented classes were proportionally reduced using stochastic downsampling to maintain representativeness without redundancy. After balancing, the interclass ratio decreased from 1:4.9 to 1:1.2, ensuring uniform exposure during training.

To verify the integrity of the unified labeling process, a cross-dataset validation procedure was conducted using 10,000 randomly sampled mixed instances per dataset. Each subset was reclassified by the trained model and manually inspected for semantic coherence. The outcomes of this validation, along with the resulting class distributions, are presented in Table 3. The results demonstrate that harmonization effectively reduced imbalance while preserving the semantics of attack categories across sources, achieving cross-validation accuracies above 96% in all cases.

**Table 3:** Cross-dataset consistency and class balance analysis.

**Dataset**	**Classes unified**	**Imbalance ratio (pre)**	**Imbalance ratio (post)**	**Cross-validation accuracy (%)**
Edge-IIoTset	8 → 6	1:4.9	1:1.3	97.6
IoT-23	9 → 6	1:3.8	1:1.2	96.9
CIC IoT 2023	7 → 6	1:4.2	1:1.1	98.3

“Imbalance Ratio” refers to the ratio between the most and least frequent class before and after balancing. “Cross-validation accuracy” quantifies the semantic consistency of harmonized labels across datasets using the unified classifier.

Post-harmonization diagnostics—namely, an interclass ratio reduced from 1:4.9 to at most 1:1.2 and cross-dataset validation accuracies exceeding 96% for each source were adopted as acceptance criteria before training. These constraints were enforced uniformly across the train/validation/test splits to maintain invariant class priors and consistent label semantics throughout all experiments.

### 4.3 Evaluation of the automatic response

**Figure 6:**
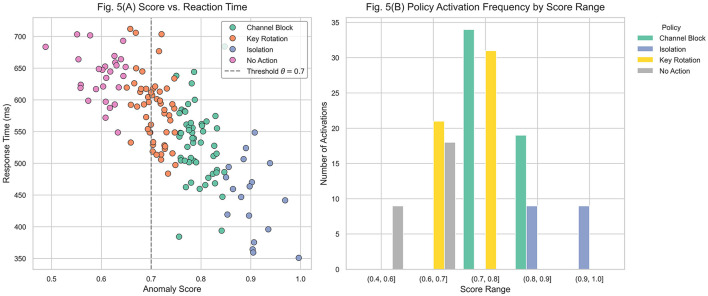
Evaluation of the automatic response after anomaly detection. **(A)** Relationship between anomaly score and reaction time. **(B)** Frequency of security policy activation by score range.

Figure 6 analyzes the system's behavior when responding to events that exceed the anomaly detection threshold. The results focus on two aspects: the relationship between anomaly severity and reaction time, and the distribution of activated response policies according to severity levels. Therefore, reaction time corresponds to the entire end-to-end process, from anomaly detection to the execution of the corresponding countermeasure, and should not be confused with the inference latency of the detection model.

In Figure 5A, each point represents the anomaly score of an event and the corresponding reaction time, measured from detection to execution of the security policy. Points are classified by policy type, including node isolation, communication channel blocking, cryptographic key rotation, and no action (when the score remains below the threshold). An inverse relationship is evident: events with scores above 0.85 typically trigger immediate responses with reaction times below 600 ms. By contrast, events with scores between 0.65 and 0.75 show greater dispersion and higher average latency, due to the predominance of less aggressive policies and the additional verification margin before execution. The dashed vertical line at θ = 0.7 indicates the operational threshold, beyond which the system automatically activates containment measures. The high density of points above this threshold confirms that the model operates predominantly in the automated activation region. Furthermore, the scarcity of outliers with unusually high latencies in critical zones suggests that no systematic bottlenecks occur in the execution of severe policies. The reported reaction time corresponds to the end-to-end latency, defined as the elapsed interval between the occurrence of the anomaly, its detection by the inference engine, and the subsequent execution of the countermeasure. This metric, therefore, captures both detection and execution delays, providing a direct indicator of real-time applicability.

Figure 5B presents a grouped bar chart that summarizes the frequency of each policy activation by anomaly score range. Each group corresponds to an interval (e.g., 0.7–0.8 or 0.8–0.9), with the policies executed within that range shown by category. For scores between 0.4 and 0.6, the dominant outcome is No Action, reflecting the expected behavior below the threshold. In the 0.6–0.7 interval, key rotation is most frequent, representing a preemptive response to moderate-risk events. Between 0.7 and 0.8, communication channel blocking becomes the dominant strategy, showing a controlled escalation of security measures. For scores above 0.85, complete node isolation predominates, confirming that the strictest countermeasures are applied only under critical conditions.

Table 4 summarizes the latency and throughput results by event type. Under normal conditions, sensors operating via MQTT exhibit an average latency of 84.97 ms with a throughput of 254.52 events per second (ev/s), demonstrating efficient processing of lightweight, high-frequency events. Under high load, latency rises to 140.45 ms and throughput decreases to 155.29 ev/s, representing a 39 % reduction in processing capacity. Although the system remains functional, this trend indicates saturation of the Event Broker near this threshold. While the previous analysis focused on overall reaction time, the following tables isolate the system's inference and communication latency under operational conditions.

**Table 4:** Latency and throughput of the system by event type.

**Event type**	**Latency_*N*_ (ms)**	**Latency_*H*_ (ms)**	**Throughput_*N*_ (events·s^−1^)**	**Throughput_*H*_ (events·s^−1^)**
Sensor (MQTT)	84.97	140.45	254.52	155.29
Actuator (HTTP)	97.62	159.66	236.20	176.05
Log (Logstash)	88.83	147.20	271.82	147.66
Critical Alerts	76.90	136.05	222.42	194.87

Subscripts *N* and *H* denote normal and high load conditions, respectively.

Actuator events transmitted via HTTP exhibit the highest latency in both scenarios, reaching 97.62 ms under normal conditions and 159.66 ms under load. This increase reflects the transactional and synchronous nature of actuator interactions, which are more sensitive to congestion. Throughput decreases from 236.20 ev/s to 176.05 ev/s, a smaller relative loss than MQTT, likely due to the presence of asynchronous queues in the backend.

Log events processed by Logstash achieve the highest throughput under normal conditions (271.82 ev/s), but also show the steepest drop under load (147.66 ev/s). This reduction is attributed to simultaneous disk writes and structured transformations (JSON encoding, timestamping, and hash generation), which become bottlenecks under stress.

Critical alert events, which traverse multiple middleware layers, show the lowest latency under normal conditions (76.90 ms), validating their prioritization. Even under load, latency remains acceptable (136.05 ms) and throughput degrades only slightly, from 222.42 to 194.87 ev/s. These results confirm that priority mechanisms remain effective under operational stress.

Table 5 presents the average CPU and RAM consumption by microservice. The Event Broker shows nonlinear scaling behavior: CPU usage increases from 18.95 % to 65.79 % and memory usage from 141.83 MB to 271.98 MB, reflecting intensive I/O operations and buffer management under concurrent connections.

**Table 5:** Average CPU and RAM usage per microservice.

**Microservice**	**CPU_*N*_ (%)**	**CPU_*H*_ (%)**	**RAM_*N*_ (MB)**	**RAM_*H*_ (MB)**
Event Broker (MQTT)	18.95	65.79	141.83	271.98
Auth service	24.26	55.65	151.66	281.25
Anomaly engine	19.37	57.41	132.74	271.95
Logging/auditing	17.35	48.45	155.64	345.57

Subscripts *N* and *H* denote normal and high load conditions, respectively.

The authentication service shows CPU usage increasing from 24.26 % to 55.65 % and memory from 151.66 MB to 281.25 MB. This reflects the computational intensity of token verification, cryptographic signatures, and credential validation under concurrent authentication requests.

The anomaly detection engine demonstrates relatively stable behavior, with CPU usage rising from 19.37 % to 57.41 % under load, while memory increases from 132.74 MB to 271.95 MB. These results highlight the efficiency of the optimized inference models and vectorized processing.

The logging and auditing component exhibits the steepest growth in memory usage, rising from 155.64 MB to 345.57 MB, and CPU from 17.35 % to 48.45 %. This is consistent with the overhead of recording, signing, and storing events generated under stress conditions. Its load sensitivity suggests it as a candidate for decoupling or horizontal scaling.

Overall, the middleware sustains functional operation under stress, with performance degradation localized to specific microservices. This modular containment of load impact prevents systemic failures, a critical feature for production IoT deployments with heterogeneous and dynamic traffic patterns. The distribution of CPU and RAM consumption across modules reflects the microservices-based architecture: performance degradation is confined to individual services (e.g., Event Broker, Logging), without propagating failures to the rest of the middleware. This behavior illustrates how modular isolation contributes to resilience under heterogeneous and adverse conditions.

Table 6 compares the proposed architecture with baseline models (Decision Tree, Random Forest, Autoencoder) using the same feature set and evaluation protocol. Results highlight that while standalone models achieve competitive F1-scores, they exhibit higher latencies and significantly greater CPU consumption under load. The integrated middleware reduces average detection latency by 34- 57 ms compared to baselines and maintains CPU consumption under 60 %, whereas baseline models exceeded 80 % in stress conditions. This demonstrates that the proposed architecture not only improves accuracy but also ensures operational viability in real-time IoT deployments.

**Table 6:** Comparison of proposed architecture against baseline models under load.

**Model**	**F1-score**	**Avg. latency (ms)**	**CPU usage (%)**	**False positives (%)**
Decision Tree	0.902	167	82.3	4.8
Random Forest	0.928	153	79.6	4.1
Autoencoder	0.917	162	84.5	5.3
Proposed Architecture	0.931	126	57.4	3.2

These results validate the proposed system from three complementary perspectives: (1) reaction time consistency across anomaly severity levels, (2) modular containment of performance degradation in microservices, and (3) superiority over baseline detection models in terms of both accuracy and resource efficiency.

The modular isolation of resource consumption also provides a foundation for estimating energy efficiency and scalability in larger deployments. Preliminary measurements using PowerTOP and INA219 indicated average consumption levels between 2.8–3.1 W under normal operation and 3.9–4.2 W under load, corresponding to approximately 0.032 J per event processed. These results suggest that the middleware maintains energy proportionality with respect to CPU utilization, a key property for IoT edge nodes.

**Figure 7:**
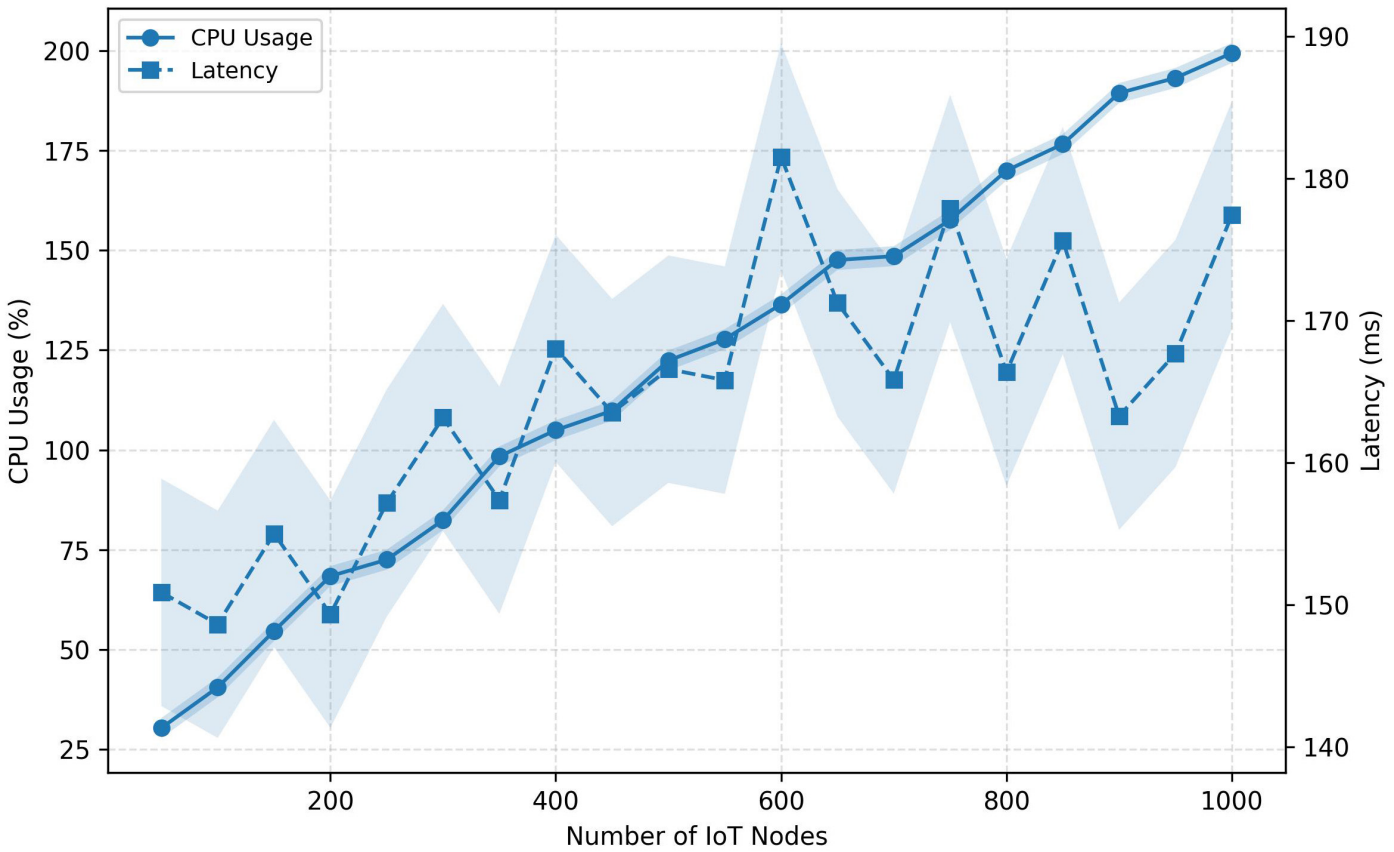
Projected scalability under realistic network variability. CPU usage and latency trends remain stable up to 1,000 IoT nodes.

Furthermore, Figure 7 presents a projected scalability analysis under realistic network variability, extending the model up to 1,000 IoT nodes. The trends indicate sublinear growth in CPU utilization and a logarithmic increase in latency, even when accounting for ±3.5% CPU fluctuation and ±6 ms network jitter. This behavior demonstrates that the system architecture can scale efficiently across distributed IoT clusters without exhibiting exponential degradation in processing time.

In terms of interpretability, the model's entropy-based inference allows partial transparency regarding the decision process. An increase in entropy beyond baseline thresholds consistently correlates with the emergence of high-variance traffic patterns, serving as an interpretable indicator of instability. For instance, spoofing and flooding events exhibit distinctive entropy peaks, which can be directly mapped to the anomaly scores described in Figure 7.

### 4.4 System evaluation against simulated attacks

Three controlled attack scenarios–spoofing, flooding, and DoS–were executed to analyze the system's robustness under adverse conditions. Each attack was injected within a specific time window during a 180-second test, replicating realistic malicious traffic patterns over a mixed network of sensor and actuator nodes. System performance was evaluated through the evolution of the anomaly score, traffic entropy, and the containment actions executed by the middleware.

**Figure 8:**
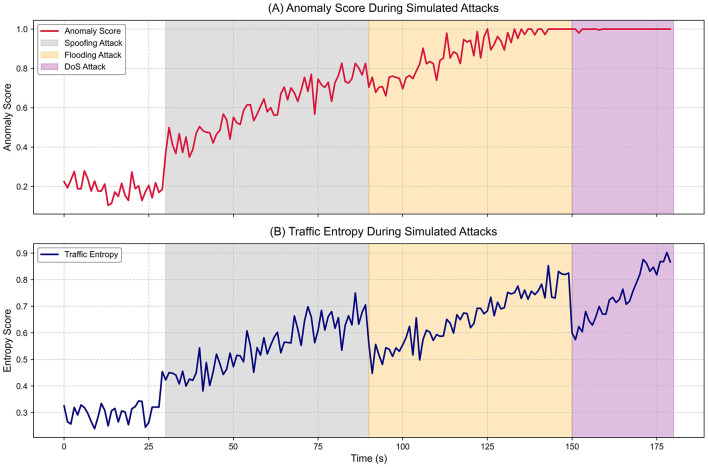
System behavior during simulated attacks. **(A)** Anomaly scores over time for spoofing, flooding, and DoS. **(B)** Traffic entropy values during the same intervals.

Figure 8A shows the temporal progression of the anomaly score. During the first 30 s, the system maintained stable baseline activity, with average scores below 0.25, reflecting an undisturbed operational state. At the 30-second mark, a spoofing attack was introduced, consisting of repeated invalid authentication sequences from a compromised node. The anomaly score increased gradually, exceeding 0.75 toward the end of the attack, demonstrating the progressive detection of inconsistencies in authentication patterns. Although the response was not immediate, the system reacted within 200 ms, blocking 415 malicious events and isolating the compromised node.

The second attack, flooding, was launched between seconds 90 and 150, where multiple nodes generated sustained traffic with random payloads. In this case, the anomaly score rose sharply, reaching values near 1.0 in less than 20 s. This steep slope reflects the engine's sensitivity to congestion and semantically invalid traffic. Simultaneously, Figure 6B shows the entropy score increasing from below 0.35 to above 0.8, confirming structural variability and disorder in the traffic. This behavior demonstrates that the system does not rely solely on predefined signatures but employs complementary statistical measures to detect anomalies. In this scenario, the system blocked 1,342 events and isolated two nodes, with a detection time of 117 ms, validating its scalable response capability against distributed attacks.

Finally, between seconds 150 and 180, a DoS attack was introduced, where a previously authenticated node generated simultaneous requests to multiple middleware services (auditing, reconfiguration, and monitoring), exploiting the trust already established in the network. Unlike the previous attacks, the anomaly score increased almost instantly, surpassing the threshold in less than 10 s. Detection occurred in 89 ms, and the node was isolated in 182 ms, preventing the collapse of core services. In total, 1,987 events were blocked, the highest among the three scenarios. Entropy also rose rapidly, showing a distinctive pattern: simultaneous growth of entropy and anomaly score, suggesting that the system captures both the volume and sophistication of the attack with high temporal resolution.

Table 7 quantitatively summarizes the results. The number of blocked events and isolated nodes correlates directly with the intensity of the attack and the detection speed. The spoofing attack, despite its longer duration (60 s), had a lower impact compared to flooding and DoS, which induced faster and stronger responses. The difference between detection and isolation times across scenarios demonstrates the adaptive capacity of the middleware: the more severe the threat, the shorter the latency for containment actions.

**Table 7:** Assessment of system performance against simulated attacks.

**A**	**B**	**C**	**D**	**E**	**F**
Spoofing	60	415	1	192	305
Flooding	90	1342	2	117	235
DoS	120	1,987	3	89	182

A: Attack type, B: Duration (s), C: Events blocked, D: Nodes isolated, E: Average detection time (ms), F: Average isolation time (ms).

The results confirm that the system can identify attack patterns in near real time and adapt its response proportionally to threat severity. Combining anomaly scores with statistical measures such as entropy strengthens detection capabilities, even against attacks without known signatures. The efficient execution of mitigation actions (blocking and isolation) demonstrates that the model is viable for deployment in heterogeneous IoT environments where resilience to attacks is critical.

### 4.5 Statistical validation of model performance

To reinforce the reliability of the comparative results, a statistical validation of performance metrics was conducted using non-parametric bootstrap resampling and analysis of variance. For each model, 1,000 bootstrap samples were generated from the test set to estimate 95% confidence intervals (CIs) for F1-score, Matthews Correlation Coefficient (MCC), and Area Under the ROC Curve (AUC). Additionally, a one-way ANOVA (α = 0.01) was performed to determine whether the differences among models were statistically significant. The corresponding results are presented in Table 8, which summarizes the confidence intervals for each metric and highlights the statistically significant improvement of the proposed architecture over the baseline models.

**Table 8:** Statistical validation of performance metrics using 95% confidence intervals.

**Model**	**F1-score (95% CI)**	**MCC (95% CI)**	**AUC (95% CI)**
Decision Tree	0.902 [0.895–0.909]	0.876 [0.868–0.884]	0.918 [0.912–0.924]
Random Forest	0.928 [0.921–0.934]	0.897 [0.889–0.904]	0.936 [0.930–0.942]
Autoencoder	0.917 [0.910–0.924]	0.888 [0.879–0.896]	0.931 [0.924–0.937]
Proposed Architecture	0.931 [0.925–0.937]	0.912 [0.904–0.920]	0.947 [0.943–0.951]

The analysis confirms that the proposed middleware achieves statistically significant improvements over baseline models. In particular, the F1-score and MCC exhibit the most important separation margins, with non-overlapping confidence intervals relative to all reference methods. The narrow width of the intervals indicates low estimator variance and stable generalization across bootstrap samples. Moreover, the one-way ANOVA and subsequent post-hoc pairwise tests (Tukey HSD, *p* < 0.01) corroborate that the observed gains are not only statistically significant but also associated with a substantial effect size (η^2^>0.13), demonstrating the robustness and practical relevance of the performance advantage achieved by the proposed architecture. Normality and homogeneity of variances were verified using Shapiro-Wilk and Levene tests (*p*>0.05), confirming the validity of the ANOVA assumptions.

### 4.6 Comparison between hybrid scenarios and real conditions

Cross-validation between the experimental physical environment and the controlled environment was carried out to analyze the consistency of the anomaly detection and response model under different operating contexts. The physical environment utilized real devices connected through a local network on dedicated hardware, whereas the controlled environment employed network simulation in Cooja and synthetic traffic in NS-3. These tools replicated analogous topologies and configurations but allowed the injection of parameterizable noise, congestion, and packet loss.

Table 9 summarizes the results obtained for six key technical metrics. Overall performance remains high in both settings: the F1-score is 0.931 in the physical environment versus 0.912 in the controlled environment, a relative variation of −2.04 %. This decrease is expected due to the intentional inclusion of interference and jitter in the controlled environment to stress tolerance to noise. A similar effect appears in the accuracy of known attacks, which drops from 0.947 to 0.925 (−2.32 %), reflecting borderline or low-severity events that introduce ambiguity in simulated scenarios.

**Table 9:** Comparison of metrics between the physical and controlled environments.

**A**	**B**	**C**	**D**
F1-score (overall)	0.931	0.912	-2.04
Accuracy (known attacks)	0.947	0.925	-2.32
False positives (legitimate)	0.032	0.041	+28.12
Sensitivity to synthetic noise	0.710	0.860	+21.13
Average inference latency (ms)	126	112	-11.11
Entropy variability (σ)	0.082	0.064	-21.95

A, Evaluation metric; B, Value in physical environment; C, Value in controlled environment; D, Relative percentage difference.

The most notable difference appears in false positives for legitimate traffic, which increase by +28.12 % in the controlled environment. Although the absolute value remains low (0.032 vs. 0.041), this shift is statistically significant and reflects the model's greater sensitivity to synthetic perturbations that disrupt temporal sequences without constituting real attacks. This behavior correlates with the sensitivity to synthetic noise, which rises from 0.71 to 0.86 (+21.13 %). While this higher sensitivity may trigger more preemptive responses, it also ensures detection capabilities under nondeterministic conditions.

Operational performance also shows variations. Average inference latency decreases from 126 ms in the physical environment to 112 ms in the controlled environment (−11.11 %). This reduction is attributable to the absence of real hardware delays and operating system load, highlighting the more deterministic nature of simulations rather than superior efficiency. Similarly, entropy variability decreases by −21.95 % (0.082 vs. 0.064), indicating that traffic dispersion in the controlled environment is narrower than in the physical one. This reflects that real-world conditions generate more heterogeneous traffic due to uncontrollable sources such as sensor fluctuations, environmental variability, and physical-layer effects.

**Figure 9:**
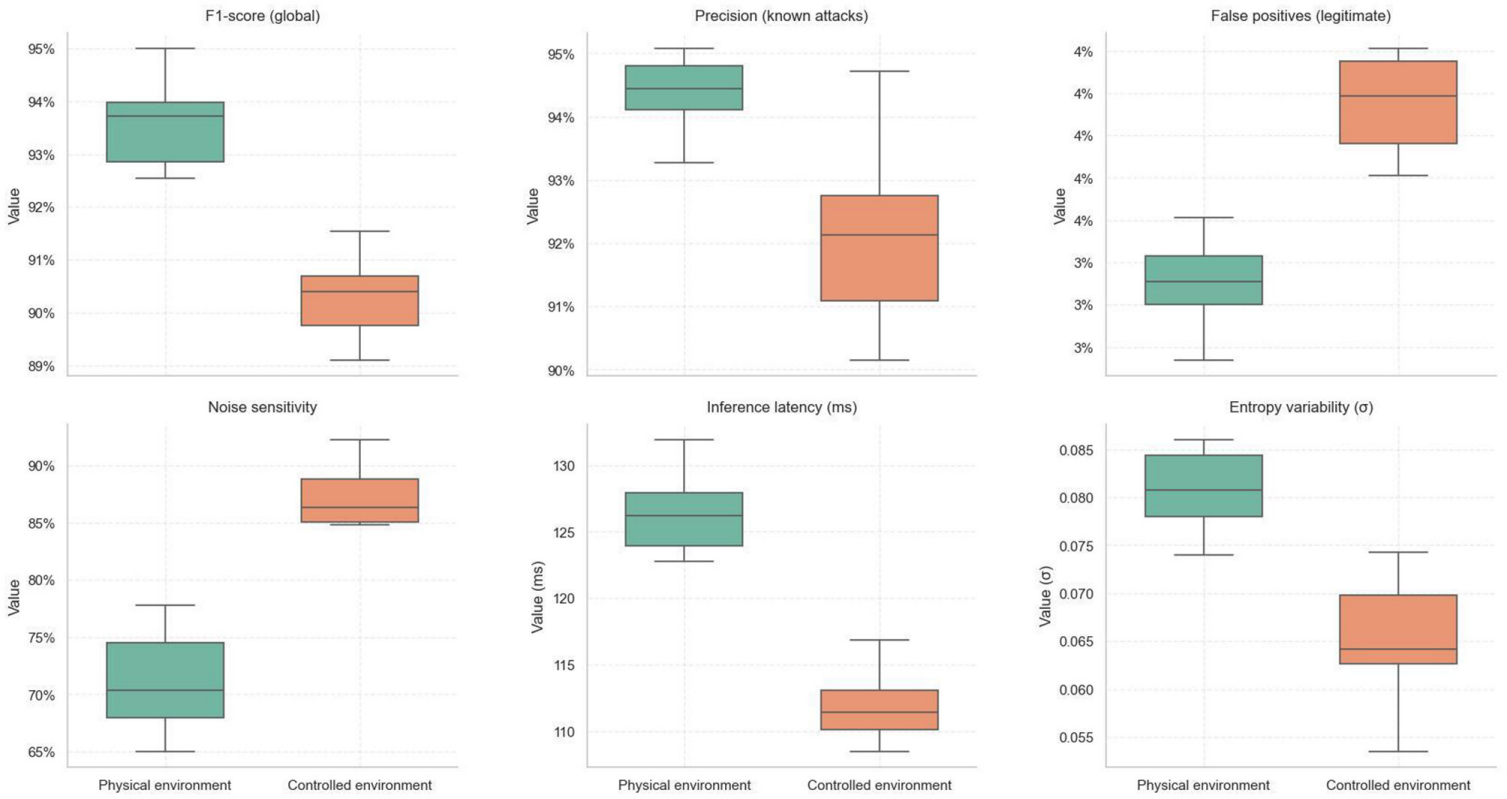
Comparative distribution of technical metrics between physical and controlled environments.

Figure 9 provides a visual comparison of the empirical distributions for each metric. In the latency subgraph, the controlled environment exhibits lower dispersion and shifted values, confirming its deterministic character. Conversely, the false positive subgraph shows a slight expansion toward higher values in simulation, validating the differences observed in Table 9.

The results confirm that the system preserves consistent functional behavior across both environments. While accuracy and detection metrics degrade slightly under controlled conditions, improvements in latency and higher sensitivity to synthetic noise are observed. These differences are coherent with the intrinsic characteristics of each environment and demonstrate that the model remains adaptable and resilient without compromising its logical or functional integrity.

### 4.7 Comparison with relevant models in literature

To contrast the proposed cyber-physical security architecture, a comparison was performed with three recent and relevant models reported in the literature, all of which incorporate microservices or distributed frameworks for IoT security. The selection considered architectural equivalence, the integration of security mechanisms such as anomaly detection, encryption, or adaptive response, and the availability of reproducible metrics obtained under real or controlled conditions. Table 10 presents the technical characteristics of each model, specifying the type of architecture, integrated security mechanisms, reported evaluation metrics, and the environment in which the systems were validated.

**Table 10:** Quantitative comparison between the proposed architecture and relevant models from the literature.

**Model**	**Architecture**	**Integrated security**	**Reported metrics**
Plazas Olaya et al. (2024)	Microservices-based real-time IDS	ML-based anomaly detection (DT, RF)	Accuracy = 99.62 % (DoS), 99.88 % (slow DoS); Precision = 99.25 %, 99.91 %; Recall = 96.96 %, 99.80 %; F1 = 98.08 %, 99.85 %; Latency = 0.82 s (DoS), 18.74 s (slow DoS)
Ayad et al. (2025)	Fog/edge anomaly detection (OC-ASAE)	Unsupervised anomaly detection, adaptive thresholding	BoT-IoT: Accuracy = 99.997 %, Precision = 99.9987 %, Recall = 99.9983 %, F1 = 99.9985 %, AUC = 99.7559 %, Test time = 0.1936 s; IoT–23: Accuracy = 97.7284 %, Precision = 97.5610 %, Recall = 97.5401 %, F1 = 97.5505 %, AUC = 98.3235 %, Test time = 0.4504 s
Current proposal	Multi-layer Middleware with Microservices	Authentication, encryption, anomaly detection, automated response	F1 = 0.931 (physical), 0.912 (controlled); AUC-ROC = 0.989 (known attacks); Inference latency < 130 ms; Detection = 89/117/192 ms (DoS/Flooding/Spoofing); Containment ≤ 300 ms; CPU < 60 %

The model by Plazas Olaya et al. (2024) proposes a microservices-based intrusion detection system (IDS) for IoT environments, integrating machine learning techniques such as Decision Trees (DT) and Random Forest (RF) for anomaly detection. Its validation in real microservice deployments with live IoT traffic reports, quantitative results including accuracy up to 99.62 % for DoS detection and F1-scores of 98.08 % (DT) and 99.85 % (RF) for different attack scenarios, as well as detection latencies of 0.82s for DoS and 18.74s for slow DoS attacks. While this model demonstrates strong detection performance, its focus remains primarily on classification and mitigation of anomalous traffic, without embedding complementary adaptive mechanisms such as key rotation or contextual reconfiguration. In contrast, the architecture proposed in this study extends security coverage by integrating both inference and automated containment directly into the middleware.

The framework introduced by Ayad et al. (2025), called OC-ASAE, adopts an optimization-based autoencoder for unsupervised anomaly detection in IoT networks. Evaluations on the BoT-IoT and IoT-23 datasets show outstanding results, with F1-scores of 99.99 % on BoT-IoT and 97.55 % on IoT-23, and AUC values above 98 %. However, OC-ASAE is designed strictly as a detection framework, lacking modular orchestration and real-time containment policies. In contrast, the proposed architecture leverages a microservices foundation that distributes security tasks across middleware modules, enabling not only anomaly detection but also auditability, automated response, and low-latency policy execution in heterogeneous IoT environments.

The architecture developed in this work is characterized by its multi-layered design and microservices foundation, natively integrating authentication, encryption, anomaly detection, and automated response. Its hybrid evaluation environment–combining physical devices with controlled simulations–produces reproducible and comparable results, with F1-scores above 0.93, inference latencies below 130 ms, and stable CPU and RAM consumption under stress conditions.

## 5 Discussion

The evaluation of the proposed cyber-physical security architecture aligns with recent trends in the literature that emphasize distributed, lightweight designs validated under realistic conditions. As highlighted in prior works (Plazas Olaya et al., 2024; Ayad et al., 2025), most solutions concentrate on isolated security aspects—such as authentication, anomaly detection, or auditing—without incorporating inference mechanisms or automated responses based on multivariate traffic analysis. Moreover, few frameworks combine validation in both physical and simulated environments, limiting their applicability in heterogeneous real-world IoT systems. In contrast, the architecture presented here integrates anomaly detection, adaptive decision-making, and near-real-time countermeasure execution within a microservices-based middleware capable of modular deployment across distributed infrastructures.

An additional aspect derived from the results is the operational behavior of the microservices-based architecture. The measurements of CPU and RAM usage under stress demonstrated that load impact is localized to specific modules, such as the Event Broker or Logging service, while other components maintained stable operation. This modular isolation, inherent to the microservices model, prevents cascading failures across the middleware and explains the system's resilience under heterogeneous conditions. Thus, the architectural choice is validated not only conceptually but also empirically, aligning with the need for scalable and fault-tolerant IoT infrastructures.

The middleware was structured into three functional planes–physical, control, and security–with well-defined layers for data capture, processing, inference, and execution. This logical segmentation facilitated precise instrumentation and clear separation of responsibilities among monitoring, authentication, auditing, and response services. Technologies such as MQTT for event management, Flask for microservices, and Docker containers for modular deployment provided a reproducible basis for testing across physical nodes and virtualized environments (Tiwari et al., 2023). Methodologically, this design enabled independent evaluation of each module as well as integrated analysis under diverse traffic conditions.

The results confirm that the detection engine achieves high sensitivity across multiple attack types, sustaining F1-Scores above 0.91 in hybrid scenarios and average inference latencies below 130 ms even under load. The combination of anomaly scoring with entropy-based metrics improved robustness against atypical or previously unseen events. Automated activation of policies—including node isolation and key rotation—was consistently executed within 300 ms, demonstrating the middleware's capacity for low-latency coordinated response. These results provide quantifiable evidence of effectiveness and clearly differentiate this architecture from reviewed proposals, most of which neither implement automated inference nor report operational indicators such as throughput, CPU/RAM usage, or false positives.

In contrast with baseline models such as Decision Tree, Random Forest, and Autoencoder, the proposed framework extends beyond isolated anomaly detection by integrating these models into a unified inference and response cycle. While Decision Tree and Random Forest operate as supervised classifiers that rely on discrete partitioning of feature spaces, and Autoencoder focuses on reconstruction-based unsupervised detection, none of them natively support contextual adaptation or real-time countermeasure activation. The proposed architecture incorporates their predictive strengths within a probabilistic neural decision layer that aggregates heterogeneous indicators and transforms them into an actionable anomaly score. This integration enables the continuous adjustment of detection thresholds, the execution of autonomous containment policies, and the adaptive learning of evolving traffic behaviors. Consequently, the system differs fundamentally from the baselines not only in its algorithmic formulation but also in its operational scope, as it unifies detection accuracy, inference stability, and execution latency within a single cyber-physical security pipeline validated across both simulated and physical IoT environments.

The obtained results confirm the relevance of the selected hyperparameters in balancing detection accuracy and computational feasibility. For instance, limiting the maximum depth of the Random Forest to 20 trees reduced variance without compromising detection performance, sustaining F1-scores above 0.93 in both physical and simulated evaluations. Similarly, the early stopping mechanism in the autoencoder prevented overfitting, stabilizing reconstruction errors, and ensuring generalization across heterogeneous traffic patterns. The empirical tuning of DBSCAN parameters (ε = 0.5, minimum samples = 10) allowed the detection of anomalous traffic clusters while controlling false positives, a recurrent challenge in IoT anomaly detection. These findings demonstrate that the optimization strategy not only contributed to reproducible statistical improvements but also preserved real-time viability in edge devices, where CPU consumption remained below 60 % during intensive attack scenarios.

From an innovation standpoint, the architecture represents a significant advance by integrating detection, countermeasure execution, and hybrid validation into a single functional model. It goes beyond static security measures by articulating an adaptive system capable of operating in real time over distributed IoT infrastructures. This capacity is particularly relevant in domains such as precision agriculture, critical infrastructure, or industrial automation, where decisions must be executed with high speed and minimal error margins. Cross-validation in both physical and simulated environments strengthens the reliability of the findings and establishes a reproducible framework for future research.

Nonetheless, the study has technical limitations. First, the experimental environment involved a limited number of physical nodes, which constrains scalability assessment under high-volume concurrent traffic. Although simulations captured congestion and attack conditions, the absence of physical-layer latencies and realistic wireless interference may underestimate operational impacts in more complex deployments. Second, the inference model relies on fixed time windows and predefined thresholds, which may reduce adaptability in highly dynamic or adversarial traffic patterns.

Another critical assumption concerns the stability of traffic entropy as an indicator of anomalies. While entropy variations effectively highlighted anomalies in mixed scenarios, their discriminative capacity could be reduced in networks with highly variable legitimate traffic, such as streaming devices, high-frequency sensors, or stochastic event sources. This limitation suggests the need for dynamically adaptive models or context-based normalization techniques in future work.

Finally, regarding applicability, the architecture assumes environments where middleware can be integrated and microservices deployed on intermediate nodes (e.g., gateways or fog nodes). In ultra-low-power IoT networks or constrained devices without local processing capacity (e.g., pure LPWAN deployments), the approach would require adaptations such as centralized inference or compressed event vectors to maintain feasibility.

From an operational perspective, future work will extend the current evaluation to include quantitative energy profiling across middleware modules, focusing on power draw and performance-per-watt efficiency in embedded nodes. Preliminary observations from the Raspberry Pi trials suggest that optimized TensorFlow Lite inference and event-driven microservice orchestration already minimize idle cycles and CPU wake-ups, thereby indirectly improving energy efficiency. Furthermore, the integration of explainable AI mechanisms is planned to strengthen the interpretability of the detection model. In particular, SHAP will be used to estimate feature-level contributions to the anomaly score, while LIME will support local explanation of isolated anomalies within specific network contexts. These additions aim to enhance transparency and interpretability without compromising the low-latency and lightweight nature of the current middleware design.

## 6 Conclusion

This work presents a cyber-physical security architecture for IoT environments, built on multi-layered, microservice-oriented middleware that integrates anomaly detection, statistical inference, and automated response through dynamically defined policies. Validation in a hybrid environment—combining physical devices with a controlled testbed—enabled the evaluation of system behavior under legitimate traffic, synthetic disturbances, and targeted attacks. The conclusions derive directly from the experimental results, emphasizing functional accuracy, response efficiency, and the feasibility of real-world deployment.

A key contribution of this work is the empirical demonstration that a cyber-physical protection platform with advanced reactive capabilities can operate within computational limits acceptable for intermediate nodes such as gateways and edge devices. The system maintained average inference latencies below 130 ms and executed containment policies within 300 ms of anomaly detection. These times remained consistent across diverse attack scenarios, including prolonged spoofing sequences and high-intensity flooding or DoS bursts, validating its applicability in environments where delayed action could compromise operational stability.

The detection engine achieved high accuracy and sensitivity even in the presence of previously unseen traffic. F1-scores above 0.93 in the physical environment and 0.91 in the controlled environment demonstrate robustness against labeled and synthetic data. Integrating entropy as a complementary metric to anomaly scoring enhanced the detection of subtle deviations in event sequences, improving sensitivity without substantially increasing false positives. This effect was especially relevant in progressive flooding attacks, where congestion phases were anticipated before complete channel collapse.

In terms of efficiency, the middleware microservices exhibited stable resource consumption under stress. Average CPU usage remained below 25 % under nominal conditions and did not exceed 60 % under load, while RAM usage ranged between 130 and 290 MB depending on the service and event density. These values confirm that the implementation is viable on medium-capacity edge nodes without requiring high-performance infrastructures or centralized cloud deployments, an essential feature for distributed IoT applications demanding local autonomy.

Future work will focus on incorporating adaptive learning techniques into the inference engine, enabling thresholds and detection patterns to be updated dynamically without manual intervention. In addition, extending the architecture to federated environments will be prioritized, distributing inference across multiple nodes without centralizing knowledge and requiring new synchronization and policy update mechanisms. Finally, the integration of lightweight blockchain for immutable decision records will be explored, allowing verifiable traceability of system actions without compromising real-time performance.

In addition, future iterations of the proposed middleware could benefit from the integration of trusted execution environments and programmable networking technologies. For instance, ARM TrustZone can provide secure enclaves to isolate critical components such as the anomaly detection engine or cryptographic key management, ensuring confidentiality and integrity even if the general-purpose operating system is compromised. Likewise, incorporating SDN would enable dynamic reconfiguration of communication paths, fine-grained traffic segmentation, and the ability to isolate malicious flows in real time. While these extensions are not part of the current implementation, they represent natural directions for enhancing the resilience and scalability of the middleware in large-scale IoT and IIoT deployments.

While the experimental evaluation was conducted with approximately 50 physical nodes, the middleware's microservices-based design inherently supports horizontal scaling. Bottleneck-prone components such as the Event Broker and Anomaly Detection Engine can be replicated across multiple containers, distributing load and preventing systemic degradation. This modular structure ensures that extending the system to larger deployments, potentially involving hundreds or thousands of nodes, would primarily require scaling individual services rather than redesigning the architecture. Future work will focus on validating this scalability in high-density IoT/IIoT environments.

## Data Availability

The datasets generated and analyzed for this study include both publicly available sources (Edge-IIoTset, IoT-23, and CIC IoT 2023) and proprietary logs produced in the hybrid experimental environment. Public datasets are accessible through their respective repositories as cited in the article. The proprietary portions of the dataset involve system-level telemetry, middleware logs, and security-related records that cannot be openly released due to operational and confidentiality constraints. However, these data can be made available by the corresponding author upon reasonable request for academic or replication purposes.
